# Evidence for conservation of a primordial 12-hour ultradian gene program in humans

**DOI:** 10.1101/2023.05.02.539021

**Published:** 2023-05-02

**Authors:** Bokai Zhu, Silvia Liu, Natalie L. David, William Dion, Nandini K Doshi, Lauren B. Siegel, Tânia Amorim, Rosemary E. Andrews, GV Naveen Kumar, Saad Irfan, Tristan Pesaresi, Ankit X. Sharma, Michelle Sun, Pouneh K. Fazeli, Matthew L. Steinhauser

**Affiliations:** 1 Aging Institute of UPMC, University of Pittsburgh School of Medicine; Pittsburgh, Pennsylvania, USA; 2 Pittsburgh Liver Research Center, University of Pittsburgh; Pittsburgh, Pennsylvania, USA; 3 Division of Endocrinology and Metabolism, Department of Medicine, University of Pittsburgh School of Medicine; Pittsburgh, Pennsylvania, USA; 4 Department of Pathology, University of Pittsburgh School of Medicine; Pittsburgh, Pennsylvania, USA; 5 Neuroendocrinology Unit, Division of Endocrinology and Metabolism, Department of Medicine, University of Pittsburgh School of Medicine; Pittsburgh, Pennsylvania, USA; 6 Center for Human Integrative Physiology, University of Pittsburgh School of Medicine; Pittsburgh, Pennsylvania, USA; 7 Division of Cardiology, Department of Medicine, University of Pittsburgh School of Medicine; Pittsburgh, Pennsylvania, USA

## Abstract

While circadian rhythms are entrained to the once daily light-dark cycle of the sun, many marine organisms exhibit ~12h ultradian rhythms corresponding to the twice daily movement of the tides. Although human ancestors emerged from circatidal environment millions of years ago, direct evidence of the existence of ~12h ultradian rhythms in humans are lacking. Here, we performed prospective, temporal transcriptome profiling of peripheral white blood cells and identified robust ~12h transcriptional rhythms from three healthy subjects. Pathway analysis implicated ~12h rhythms effecting RNA and protein metabolism, with strong homology to the circatidal gene programs previously identified in *Cnidarian* marine species. We further observed ~12h rhythms of intron retention events of genes involved in MHC class I antigen presentation in all three subjects, synchronized to those of mRNA splicing gene expressions in each individual. Gene regulatory network inference revealed XBP1, GABPA and KLF7 as putative transcriptional regulators of human ~12h rhythms. These results thus establish human ~12h biological rhythms have a primordial evolutionary origin and are likely to have far-reaching implications in human health and disease.

## Introduction:

Biological rhythms are conserved from single-cell organisms to humans. The relevance of biological rhythms is best exemplified by circadian rhythms, which are entrained to a ~24-hour cycle by the rotation of the Earth and daily exposure to sun light. Circadian gene expression programs in turn regulate canonical hormonal systems, vasomotor activity, coagulation, amongst other processes, accounting for predilection of certain disease events at specific times of day [1]. Genetic variants in core circadian genes and disruptions of circadian rhythms are both associated with increased risks of many human diseases [1–4]. Thus, the wide-ranging pathological effects due to circadian disruption demonstrates the centrality of the ~24h clock to normal homeostasis and disease pathobiology [1].

Our understanding of biorhythms is now expanded by discoveries of alternative, noncircadian rhythms in lower organisms. Coastal marine organisms, such as the sea anemone *A. diaphana,* exhibit ~12h ultradian rhythms, entrained by the twice daily movements of the tides [5]. Oscillation of some physiological metrics at an approximate 12h interval in humans suggests the possibility of a corresponding ~12h molecular oscillator [6]. However, direct evidence for ~12h rhythms at the molecular level in humans are lacking. In this study, we tested whether ~12h ultradian rhythms are conserved in humans. We performed a prospective longitudinal study of three healthy subjects admitted to an inpatient clinical research unit for high temporal resolution (q2h) sampling of peripheral white blood cells. We discovered a shared ~12h gene program with homology to our ancient marine ancestors and involving fundamental pathways of protein and RNA metabolism. Additionally, we uncovered a previously unappreciated role of mammalian ~12h rhythms in regulating the splicing fidelity of immune-related mRNAs.

## Results:

### Identification of a ~12h gene program in human white blood cells

**Fig. 1.**
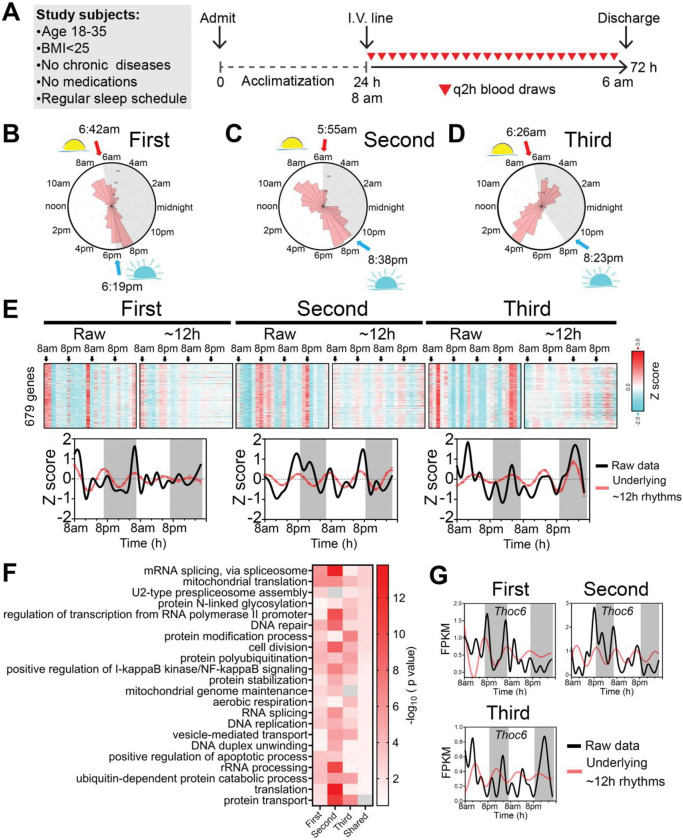
Discovery of a ~12h transcriptome involved in mRNA and protein metabolism. (**A**) Human protocol and study schematic. (**B-D**) Polar histograms showing the phase distributions of ~12h rhythms in the first (**B**), second (**C**) and third (**D**) subjects. The times of sunrise and sunset during sample collection are indicated. (**E**) Heatmap demonstrating expression of 679 ~12h genes common to all three subjects, with raw expression and superimposed ~12h rhythms shown. All data are Z score normalized. (**F**) GO analyses of ~12h genes for each subject and the ~12h genes common to all subjects (shared). (**G**) Temporal expression of *THOC6* in all three subjects.

We studied 3 healthy male subjects (Table 1) who did not engage in nighttime shift work or other routine nighttime sleep-disrupting activities, who self-reported a regular nighttime sleep schedule, and who had a body mass index between 18.5–24.9 kg/m^2^. Subjects were admitted for a 1-day acclimatization period prior to the 48 hours of blood sampling at 2-hour sampling interval (Fig. 1A). We selected the 2-hour sampling frequency to have sufficient power to detect oscillations with periods larger than 4 hours with high confidence (the Nyquist frequency of 2-hour sampling frequency is 4-hours) [7, 8]. This *a priori* protocol design reflected our aim to sample with sufficient frequency and duration to detect ~12h rhythms within any given individual. We also designed the protocol to otherwise resemble the subjects’ free-living conditions by feeding them a diet similar in caloric content and macronutrient composition to their typical intake and encouraging maintenance of their routine sleep/wake cycles. Overnight, blood was collected via a long intravenous line from outside the room to avoid exposing subjects to light or sleep disruption.

We performed bulk mRNA-Seq in buffy coat fractions prospectively collected at 2h intervals for 48 hours (24 samples/subject) (Fig. 1). We performed spectrum analysis with the eigenvalue/pencil method [8–13], which unlike statistical methods such as JTK_CYCLE and RAIN does not require pre-assignment of period range and thus allows unbiased identification of multiple oscillations for any given gene [8–13]. Indeed, the temporal expression of most genes was comprised of superimposed oscillations of varying periods (figs. S1 to 3) with harmonic dynamics most apparent for the oscillation with the largest amplitude (figs. S1A and S2A). While each subject demonstrated circadian rhythmicity (fig S4), we also detected a robust ~12h gene program in all three subjects. A subset of genes common to all three subjects (n=679) exhibited ~12h periodicity (Fig. 1, B to E). Since the eigenvalue/pencil algorithm is a non-statistical signal processing method, we used a permutation-based method that randomly shuffles the time label of gene expression data to determine the false discovery rate (FDR) for the ~12h gene list [11, 14]. The FDR for the ~12h gene lists was estimated to be 0.33, 0.36 and 0.38 for each of the three subjects respectively (figs. S1F, S2F, and S3F), a range in line with prior studies of circadian rhythms [14–16]. The calculated FDR is likely an overestimation since amplitude and phase information for the ~12h rhythms is not considered in the permutation dataset. ~12h gene expression oscillated with amplitudes between 2 to 8-fold (figs. S1E, S2E and S3E), was dampened in the second day in the first subject but remained steady over the 48 hours of the study period in the second and third subjects (Fig. 1E). ~12h rhythms peaked around 8am and 8pm in the first and second subjects (Fig. 1, B and C, figs. S1C and S2C), and around 4am and 4pm in the third (Fig. 1D and fig. S3C). These times largely coincide with the two ‘rush hours’ of the day at dawn and dusk. The representation of biological pathways in the ~12h gene sets was even more similar across the three subjects relative to specific ~12h gene lists. In all three individuals, ~12h genes were strongly enriched in mRNA and protein metabolism pathways, with mRNA splicing (such as *THOC6*) and translation (such as *MRPL27*) among the top enriched processes (Fig. 1, F and G and figs. S1J, S2J, S3J and S5, A and B).

To assess the robustness of the uncovered ~12h gene program, we applied an orthogonal rhythm-identification algorithm, RAIN [17], which detects rhythms with arbitrary waveforms exhibiting a single pre-specified period [8, 9, 11]. We inputted each temporal transcriptomic dataset as a continuous 48h time series dataset with a single data point at each time point (RAIN conti) or as a 24h time series dataset where two data points collected at the same time on two consecutive days were treated as biological replicates (RAIN dupli). The latter biological duplicate analytical approach revealed a larger ~12h gene programs (p value cut-off of 0.05) with lower FDRs: 3,462 genes (FDR=0.224), 7,060 genes (FDR=0.119) and 4,807 genes (FDR=0.166) (figs. S6 and 7). Regardless of input, the RAIN method identified ~12h rhythms in gene expression enriched in pathways related to mRNA and protein metabolism and concordant with the eigenvalue/pencil method (figs. S6 and 7). These data collectively demonstrate ~12h rhythmic gene programs with unique functions related to RNA and protein metabolism in humans.

### XBP1, GABPA and KLF7 are putative transcriptional regulators of human ~12h rhythms.

**Fig. 2.**
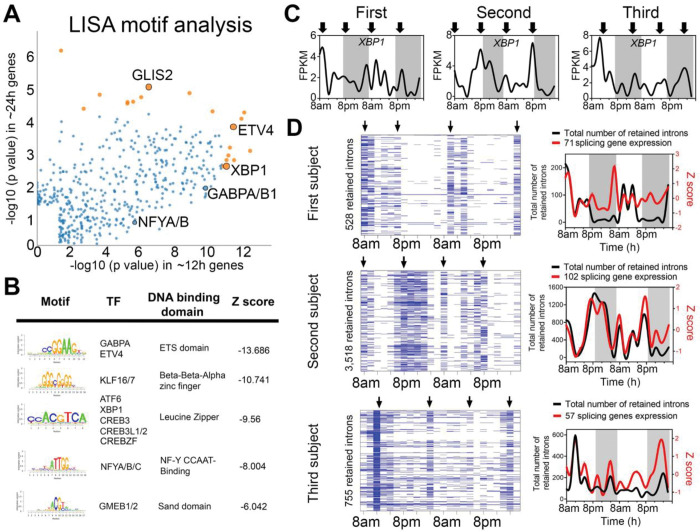
Regulatory and functional dissection of human ~12h rhythms. (**A**) Scatter plot demonstrating the log normalized p values of identified motifs for the 679 common ~12h genes (x axis) versus the 66 common circadian genes using the LISA program. ETV4, XBP1, GABPA and NYF motifs are highly represented at the promoters of ~12h genes. Orange color indicate top ten motifs associated with ~12h and 24h genes. (**B**) Top motifs enriched at the promoters of ~12h genes using the SeqPos motif tool in Galaxy/Cistrome. (**C**) Temporal expression of XBP1 in all three subjects. (**D**) Heatmap (left) and quantification (right) of temporal intron retention events, superimposed with the Z score normalized temporal expression of splicing genes exhibiting ~12h rhythms.

To infer gene regulatory networks governing ~12h rhythms, we performed LISA [18] and motif analysis on the common ~12h gene program. We cross-referenced enriched motifs with transcription factor genes that exhibited ~12h rhythms, identifying XBP1, GABPA, NFYB, ETV4, GLIS2 and KLF7 as top candidates (Fig. 2, A to C). Among them, *XBP1*, *GABPA* and *KLF7* met a higher level of stringency having been identified by both the eigenvalue and RAIN methods. We then performed an analysis of a recently published XBP1 ChIPmentation dataset in mouse T helper cells [19] and found XBP1 chromatin binding at the promoter regions of 62 murine orthologs contained in the subset of the high stringency ~12h genes with mouse orthologs (n=93 orthologs), including mRNA processing genes *Thoc6*, *Smu1*, *Snrnp53*, *Eny2* and *Snrpc*, as well as *Xbp1* itself, *Gabpa* and *Klf7* (fig. S8). These results suggest XBP1, GABPA and KLF7 as candidate transcriptional regulators of ~12h rhythms in human white blood cells, with XBP1 representing a particularly strong candidate given its previously identified role as a core regulator of ~12h transcriptional oscillations in the murine liver [11].

### ~12h rhythms are synchronized to RNA splicing functionality.

Given the robust representation of RNA metabolism and mRNA splicing pathways in ~12h transcriptional rhythms, we tested whether such oscillations would translate into a downstream functional effect in the form of alterations in mRNA splicing. We interrogated the RNA-seq data for evidence of rhythmicity in intron retention (IR) events, predicting that if the rhythm of RNA splicing genes’ transcription was functionally relevant, it would temporally correlate with global IR. We applied a recently published algorithm iREAD [20]. Using two different criteria for defining retained introns, we identified ~12h rhythms of global IR in all three subjects (Fig. 2D and fig. S9). IR rhythms were highly synchronized to the expression of mRNA splicing genes (Fig. 2D and fig. S9). These data suggest synchronization of mRNA splicing gene programs to splicing functionality, further implicating ~12h rhythms in the regulation of RNA metabolism.

We next performed GO analysis on the set of transcripts in which we detected retained introns. While the GO terms associated with intron retention genes were largely consistent at the morning and evening peaks, we also observed differential enrichment in a subset. The morning intron retention gene sets were enriched in immune functions, especially those involving the display of intracellular peptide fragments to cytotoxic T cells via MHC class 1 complexes (*e.g.* HLA-A/C/E and B2M), and whereas the evening intron retention gene sets exhibited greater heterogeneity across the three subjects (figs. S10 and S11). Taken together, these data suggest that one function of human ~12h rhythms in peripheral leukocytes might be the anticipatory splicing of antigen presentation genes.

### Evolutionary conservation of the ~12h gene program and function

**Fig. 3.**
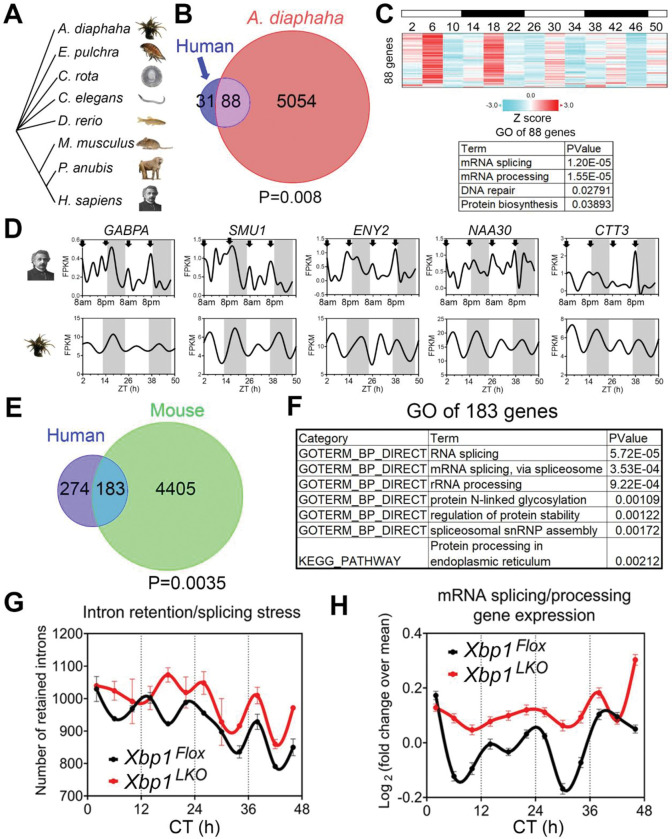
Evolutionary conservation of a ~12h gene program. (**A**) Phylogenetic tree of select species for which ~12h rhythms of gene expression have been demonstrated. Of those shown, A. diaphaha is the most distant from *H. sapiens*. (**B**) Venn diagram comparing distinct and shared ~12h genes in human and *A. diaphana*. Out of 679 genes with ~12h rhythms detected in all three human subjects, only 119 genes have orthologs that are detected in *A. diaphana*. Out of these 119 genes, 88 (74%) exhibit circatidal rhythms, statistically significantly higher (p=0.008) than the overall 62% of circatidal rhythms observed in *A. diaphana* transcriptome, indicating evolutionary conservation of 12h gene programs. (**C**) Heatmap of temporal expression (Z score normalized) of 88 circatidal genes in *A. diaphana* (top) and GO analysis of the top enriched pathways (bottom). (**D**) Representative temporal expression of select ~12h genes for human (top, second subject) and *A. diaphana* (bottom). Arrows indicate peak of expression. (**E**) Venn diagram comparing distinct and shared ~12h genes in human and mouse. 457 of the common human ~12h genes have orthologs that are detected in mice, of which 183 (40%) exhibited ~12h rhythms in murine liver, higher than expected by chance (p=0.0035). (**F**) GO analysis of 183 ~12h genes common to mice and humans. 12h rhythms of intron retention events (**G**) and RNA splicing genes (**H**) are attenuated by liver specific loss of function of XBP1 (*Xbp1^LKO^*: red), the putative master regulator of 12h gene expression oscillations, relative to control (*Xbp1^Flox^*: black).

The ~12h rhythms in coastal and estuarine animals suggests an ancient evolutionary origin [6, 21, 22] (Fig. 3A). To determine whether the hitherto identified ~12h rhythms of gene expression in humans share homology with circatidal genes, we compared the ~12h genes common to all three subjects to the circatidal gene program in *Aiptasia diaphana* [23], a sea anemone species which shares a eumetazoan ancestor with *Homo sapiens* ~700 million years ago in the Cryogenian period [24]. In line with our human data, the most enriched biological pathways in the *A. diaphana* circatidal transcriptome are mRNA processing and translation [25]. We also found 88 putative ~12h genes that overlapped with our human ~12h genes (p=0.008), a subset that was also enriched in mRNA splicing and translation pathways (Fig. 3, B to D) and included the *A. diaphana* ortholog of *GABPA* (Fig. 3D). These collective data indicate evolutionary conservation of a ~12h gene program in humans related to RNA metabolism.

If the ~12 hour gene program is conserved from coastal invertebrates to humans, we reasoned that lower mammals like mice should also exhibit oscillations in similar gene programs. We re-examined a previously published data set of temporal hepatic RNA-seq in mouse liver and found a common set of ~12h genes (n=183, p=0.0035), again highly enriched in mRNA splicing and protein processing pathways (Fig. 3E, F). To test for synchronization between the ~12h rhythms and RNA splicing in mice, we performed intron retention analysis [11]. Similar to our human data (Figure 2D), we observed strong alignment between ~12h rhythms in splicing gene expression and global intron retention events peaking at the two ‘rush hours’ (CT2 and CT14) in mice (Figure 3G, H). In addition, liver-specific genetic ablation of XBP1 (the major transcriptional regulator of murine hepatic ~12h oscillations) abolished ~12h rhythms of splicing gene expression and intron retention rhythms, leading to increased intron retention events across the day (Figure 3G, H). These collective data indicate evolutionary conservation of a ~12h gene program in humans related to RNA metabolism.

## Discussion:

In this study, we discovered a ~12h regulatory network in healthy humans, controlling gene programs related to fundamental processes of mRNA and protein metabolism. Remarkably, we found that conservation of ~12h rhythms in humans extends to specific gene orthologs and functional pathways present in marine animals. Our discovery of one such conserved ~12h pathway, ‘mRNA splicing,’ provided opportunity to establish functional significance through identification of corresponding ~12h rhythms in global intron retention events. Due to the presence of ~12h ultradian rhythms of intron retention rhythms and the synchronization of such rhythms with those of mRNA splicing gene expression in both humans and mice, we hypothesize the existence of ~12h stress fluctuations that negatively impact global mRNA splicing efficiency, a term we coin as “splicing stress”. Just as proteotoxic stress increases unfolded/misfolded proteins and the induction of the unfolded protein response (UPR) and/or the integrated stress response (ISR), we speculate that “splicing stress” accounts for the elevation of unspliced/retained introns. Consequently, this may activate an adaptive stress response (orthologous to URP and ISR) to ameliorate the original stress through increased expression of mRNA processing genes. Future efforts are needed to identify underlying mechanisms of the putative mRNA splicing stress response, and whether the ~12h oscillator may couple both proteotoxic and mRNA splicing stress responses.

We further demonstrate conservation of a potential master ~12h transcriptional regulator, *XBP1,* which regulates ~12h gene oscillations in mice [11]. *XBP1* not only exhibited ~12h rhythmicity in our human subjects but was also implicated by gene regulatory network inferences as a lead candidate regulator of human ~12h gene programs. What adaptive advantage might ~12h rhythms of gene expression confer? We reason that the ancient ~12h rhythms that evolved in marine species in response to tidal cues have been coopted by humans as an adaptive response to accommodate physiological transitions related to feeding, physical activity, and sleep that are temporally concentrated at dawn and dusk.

Interestingly, pathway analyses of rhythmic intron retention suggests that one functional consequence of ~12h rhythms in human white blood cells could be rhythmic antigen presentation by splicing of MHC class I gene transcripts. Antigen presentation by MHC class I molecules depends on the ER and Golgi apparatus [26] - whose corresponding gene programs also exhibit ~12h rhythms—suggesting that human ~12h ultradian rhythms may regulate the efficiency of antigen presentation, a process of relevance to cancer immunology [27]. Congruent with this notion are the known roles of XBP1 and IRE1α (its upstream activator) in controlling dendritic cell homeostasis, antigen presentation efficiency and cancer progression in pre-clinical mouse models [28, 29]. Future studies are needed to determine whether antigen presentation and associated immune functions are modulated by ultradian rhythms.

Similar studies of molecular rhythms in model organisms typically require terminal tissue collection and therefore require inclusion of several animals at each time point [9, 11], in which case each animal contributes data for a single time point. Given the genetic and environmental variability of free-living humans, analogous human studies have required hundreds of subjects [30]. Our study strength owes to the prospective, repeated sample collection at high temporal resolution, which enabled high fidelity testing for a ~12h gene program *within* each subject. The likelihood that rhythms uncovered in our study are functionally relevant is enhanced by the commonality of the putative ~12h pathways across all three human subjects and their conservation in organisms ranging from marine animals to humans. While these data provide evidence in support of a ~12h gene program in humans, larger studies will be needed to determine how variable the ~12h programs are in the human population, the degree to which they are sensitive to aging or environmental stressors as observed for circadian rhythms, and most importantly whether disruption of ~12h rhythms is a causal determinant of disease pathobiology. Aside from the identification of ~12h pathways related to fundamental cellular processes, several pieces of evidence suggest that ~12h rhythms are important for maintenance of homeostasis: (i) the ~12h gene program was similar in magnitude to the circadian program; and (ii) prior human studies suggest ~12h rhythms in physiological metrics of relevance to human health and disease, including heart rate variability, blood pressure, hormone levels, and cognitive function [6, 31]. As such, our discovery of ancient ~12h gene programs in humans provides rationale for future studies to determine whether ~12h rhythms should be viewed alongside circadian rhythms as a core molecular determinant of health and disease.

## Materials and Methods:

### Human subjects and study protocol

The study protocol was approved by the University of Pittsburgh Institutional Review Board (Study 20020034; approval date: 6/4/2020) and written consent was obtained from all study participants. We studied 3 healthy male subjects, who were recruited through online advertisements. Inclusion criteria consisted of individuals 18–35 years of age with a self-reported regular nighttime sleep schedule and a body mass index between 18.5–24.9 kg/m^2^. Subjects were excluded if they admitted to nighttime shift work or other regular nighttime sleep-disrupting activities, if they had any chronic medical conditions, took any medications or recreational drugs, or used tobacco products. Potential subjects presented for a screening visit, inclusive of measurement of body weight, height, BMI, and laboratory studies, including a comprehensive metabolic panel (electrolytes, kidney function and liver function tests), complete blood count, 25-OH vitamin D, and thyroid stimulating hormone level, to screen for potential subclinical chronic diseases. We excluded subjects with low hemoglobin/hematocrit, abnormal thyroid function and individuals with 25-OH vitamin D < 20 ng/mL.

Qualifying study participants maintained a food diary, which was used to estimate their daily caloric intake and subsequently presented to the University of Pittsburgh Medical Center (UPMC) Clinical Translational Research Center (CTRC) for a 3-day inpatient visit. On the morning of admission, subjects selected items from a food menu designed to match their standard daily caloric intake with a uniform macronutrient composition of 55% carbohydrates, 25% fat, 20% protein per day. No interventions were performed during the first 24-hour period of acclimatization.

On the morning of the second day at 8am, an intravenous (IV) line was placed. Blood samples were then collected every two hours for 48 hours (total 24 samples). Nighttime blood collection was performed through a long IV line from outside the room so that the study participant would not be exposed to light or woken up during blood collection. Blood was then immediately processed in the Center for Human Integrative Physiology, two floors above the UPMC CTRC by a rotating study team, all of whom were trained in the processing procedures for this study. Blood was centrifuged (1900 RCF × 10 min) and the buffy coats were collected and immediately snap frozen in liquid nitrogen for storage at −80C.

### RNA-Seq and data analysis

RNA was isolated from peripheral blood buffy coat samples on the automated Chemagic 360 (Perkin Elmer) instrument according to the manufacturer’s instructions. Extracted RNA was quantitated by Qubit^™^ RNA BR Assay Kit (Thermo Fisher Scientific) followed by the RNA quality check using Fragment Analyzer (Agilent). For each sample, RNA libraries were prepared from 100ng RNA using the KAPA RNA HyperPrep Kit with RiboErase (Kapa Biosystems) according to manufacturer’s protocol, followed by quality check using Fragment Analyzer (Agilent) and quantification by qPCR (Kapa qPCR quantification kit, Kapa biosystem) on the LightCycler 480 (Roche). The libraries were normalized and pooled, and then sequenced using NovaSeq6000 platform (Illumina) to an average of 40M 101bp paired end reads per sample. Low-quality reads and adapter sequences were trimmed from the raw sequencing data with Trimmomatic [32]. The remaining reads were then aligned to human reference genome hg38 with STAR aligner [33]. Gene counts were quantified with the STAR --quantMode GeneCounts function. Fragments per kilobase of transcript per million mapped fragments (FPKM) were quantified with Cufflinks [34].

### Identification of the oscillating transcriptome

Averaged FPKM values at each time were used for cycling transcripts identification. Lowly expressed transcripts were removed by calculating the background expression in each subject using the average expression of a panel of 62 genes known not to be expressed in peripheral blood cells. Temporal transcriptomes were subject to linear detrend prior to identification of oscillations by either the eigenvalue/pencil or RAIN methods. For the eigenvalue/pencil method [8, 9], a maximum of four oscillations were identified for each gene. Criterion for circadian genes were: period between 20h to 25h for first and second subjects and 24h to 30h for the third subject, decay rate between 0.8 and 1.2; for ~12h genes: period between 9.6h to 13.6h for the second and third subjects and 10h to 14h for the first subject, decay rate between 0.8 and 1.2; for ~8h genes: period between 6h to 8h for the first subject and 7h to 9h for the second subject, decay rate between 0.8 and 1.2; for ~16h genes; period between 14h to 18h for the third subject. The relative amplitude was calculated by dividing the amplitude by the mean expression value for each gene. To determine FDR, we used a permutation-based method that randomly shuffles the time label of gene expression data and subjected each permutation dataset to the eigenvalue/pencil method applied with the same criterion [14]. These permutation tests were run 5,000 times, and FDR was estimated by taking the ratio between the mean number of rhythmic profiles identified in the permutated samples (false positives) and the number of rhythmic profiles identified in the original data. Analyses were performed in MatlabR2017A. RAIN analysis was performed as previously described in Bioconductor (3.4) (http://www.bioconductor.org/packages/release/bioc/html/rain.html) with either 48h continuous data or 24h data with biological duplicates as input [17]. We included genes exhibiting a period between 10h and 14h with a p value less than 0.05 as having ~12h expression in all three subjects. FDR was calculated using the Benjamini-Hochberg procedure. Heat maps were generated with Gene Cluster 3.0 and TreeView 3.0 alpha 3.0 using Z score normalized values.

### Defining oscillating genes

The eigenvalue method can detect multiple superimposed oscillations. Therefore, we defined a circadian gene as one that exhibited a circadian rhythm, regardless of its amplitude relative to other superimposed oscillations. Similar criteria were applied to other oscillations. As such, a gene can meet criteria for both a circadian and ~12h gene. By comparison, we define a dominant circadian gene as one in which the superimposed circadian rhythm has the largest amplitude among all oscillations. With this definition, dominant circadian and dominant 12h genes are mutually exclusive.

### Intron retention detection

Intron retention events were detected by tool iREAD [20]. Intron retention events are selected either with default settings T>=20, J>=1, FPKM>=2 and NE score>=0.9 or more stringent settings where T>=20, J>=1, FPKM>=3 and NE score>=0.9.

### Gene ontology analysis

DAVID (Version 2021) [35] (https://david.ncifcrf.gov) was used to perform Gene Ontology analyses. Briefly, gene names were first converted to DAVID-recognizable IDs using Gene Accession Conversion Tool. The updated gene list was then subject to GO analysis using all Homo Sapiens as background and with Functional Annotation Chart function. GO_BP_DIRECT, KEGG_PATHWAY or UP_KW_BIOLOGICAL_PROCESS were used as GO categories. Only GO terms with a p value less than 0.05 were included for further analysis.

### Motif analysis

Motif analysis was performed with the SeqPos motif tool (version 0.590) embedded in Galaxy Cistrome using all motifs within the homo sapiens reference genome hg19 as background. LISA analysis was performed using webtool (http://lisa.cistrome.org/).

## Supplementary Material

Supplement 1

## Figures and Tables

**Table 1.** Subject characteristics.

	Subject 1	Subject 2	Subject 3

Age (years)	20	22	28
Body mass index (kg/m^2^)	23.3	23.7	24.4
Vitamin D (ng/mL)	85	26	22
Glucose (mg/dL)	73	84	74
BUN (mg/dL)	10	18	14
Creatinine (mg/dL)	0.9	1.0	0.9
AST (U/L)	22	41	22
ALT (U/L)	10	28	22
TSH (mIU/L)	1.306	1.48	0.803

## Data Availability

All raw and processed sequencing data generated in this study have been submitted to the NCBI Gene Expression Omnibus (GEO; http://www.ncbi.nlm.nih.gov/geo/) under accession numbers GSE220120.

## References

[1] Bass J, Takahashi JS. Circadian integration of metabolism and energetics. Science. 2010;330(6009):1349–54. doi:10.1126/science.1195027. 21127246 PMC3756146

[2] Allada R, Bass J. Circadian Mechanisms in Medicine. New England Journal of Medicine. 2021;384(6):550–61. doi:10.1056/NEJMra1802337. PMC8108270 33567194

[3] Czeisler CA, Johnson MP, Duffy JF, Brown EN, Ronda JM, Kronauer RE. Exposure to Bright Light and Darkness to Treat Physiologic Maladaptation to Night Work. New England Journal of Medicine. 1990;322(18):1253–9. doi:10.1056/nejm199005033221801. 2325721

[4] Sack RL. Jet Lag. New England Journal of Medicine. 2010;362(5):440–7. doi:10.1056/NEJMcp0909838. 20130253

[5] Andreatta G, Tessmar-Raible K. The Still Dark Side of the Moon: Molecular Mechanisms of Lunar-Controlled Rhythms and Clocks. J Mol Biol. 2020;432(12):3525–46. doi:10.1016/j.jmb.2020.03.009. 32198116 PMC7322537

[6] Ballance H, Zhu B. Revealing the hidden reality of the mammalian 12-h ultradian rhythms. Cellular and Molecular Life Sciences. 2021. doi:10.1007/s00018-020-03730-5. PMC8407301 33449146

[7] Zhu B, Dacso CC, O’Malley BW. Unveiling “Musica Universalis” of the Cell: A Brief History of Biological 12-Hour Rhythms. J Endocr Soc. 2018;2(7):727–52. Epub 2018/07/07. doi:10.1210/js.2018-00113. 29978151 PMC6025213

[8] Antoulas AC, Zhu B, Zhang Q, York B, O’Malley BW, Dacso CC. A novel mathematical method for disclosing oscillations in gene transcription: A comparative study. PLoS One. 2018;13(9):e0198503. Epub 2018/09/20. doi:10.1371/journal.pone.0198503. 30231032 PMC6145530

[9] Zhu B, Zhang Q, Pan Y, Mace EM, York B, Antoulas AC, A Cell-Autonomous Mammalian 12 hr Clock Coordinates Metabolic and Stress Rhythms. Cell Metab. 2017;25(6):1305–19 e9. doi:10.1016/j.cmet.2017.05.004. 28591634 PMC5526350

[10] Meng H, Gonzales NM, Lonard DM, Putluri N, Zhu B, Dacso CC, XBP1 links the 12-hour clock to NAFLD and regulation of membrane fluidity and lipid homeostasis. Nature Communications. 2020;11(1):6215. doi:10.1038/s41467-020-20028-z. PMC7718229 33277471

[11] Pan Y, Ballance H, Meng H, Gonzalez N, Kim S-M, Abdurehman L, 12-h clock regulation of genetic information flow by XBP1s. PLOS Biology. 2020;18(1):e3000580. doi:10.1371/journal.pbio.3000580. 31935211 PMC6959563

[12] Dion W, Ballance H, Lee J, Pan Y, Irfan S, Edwards C, Four-dimensional nuclear speckle phase separation dynamics regulate proteostasis. Science Advances. 2022;8(1):eabl4150. doi:doi:10.1126/sciadv.abl4150. 34985945 PMC8730402

[13] Meng H, Gonzales NM, Jung SY, Lu Y, Putluri N, Zhu B, Defining the mammalian coactivation of hepatic 12-h clock and lipid metabolism. Cell Reports. 2022;38(10):110491. doi:10.1016/j.celrep.2022.110491. 35263593 PMC8958721

[14] Rey G, Milev NB, Valekunja UK, Ch R, Ray S, Silva Dos Santos M, Metabolic oscillations on the circadian time scale in Drosophila cells lacking clock genes. Molecular systems biology. 2018;14(8):e8376. Epub 2018/08/04. doi:10.15252/msb.20188376. 30072421 PMC6078164

[15] Robles MS, Cox J, Mann M. In-vivo quantitative proteomics reveals a key contribution of post-transcriptional mechanisms to the circadian regulation of liver metabolism. PLoS Genet. 2014;10(1):e1004047. doi:10.1371/journal.pgen.1004047. 24391516 PMC3879213

[16] Mauvoisin D, Wang J, Jouffe C, Martin E, Atger F, Waridel P, Circadian clock-dependent and -independent rhythmic proteomes implement distinct diurnal functions in mouse liver. Proc Natl Acad Sci U S A. 2014;111(1):167–72. Epub 20131216. doi:10.1073/pnas.1314066111. 24344304 PMC3890886

[17] Thaben PF, Westermark PO. Detecting rhythms in time series with RAIN. J Biol Rhythms. 2014;29(6):391–400. doi:10.1177/0748730414553029. 25326247 PMC4266694

[18] Qin Q, Fan J, Zheng R, Wan C, Mei S, Wu Q, Lisa: inferring transcriptional regulators through integrative modeling of public chromatin accessibility and ChIP-seq data. Genome Biology. 2020;21(1):32. doi:10.1186/s13059-020-1934-6. 32033573 PMC7007693

[19] Pramanik J, Chen X, Kar G, Henriksson J, Gomes T, Park J-E, Genome-wide analyses reveal the IRE1a-XBP1 pathway promotes T helper cell differentiation by resolving secretory stress and accelerating proliferation. Genome Medicine. 2018;10(1):76. doi:10.1186/s13073-018-0589-3. 30355343 PMC6199730

[20] Li H-D, Funk CC, Price ND. iREAD: a tool for intron retention detection from RNA-seq data. BMC Genomics. 2020;21(1):128. doi:10.1186/s12864-020-6541-0. 32028886 PMC7006120

[21] Castellana S, Mazza T, Capocefalo D, Genov N, Biagini T, Fusilli C, Systematic Analysis of Mouse Genome Reveals Distinct Evolutionary and Functional Properties Among Circadian and Ultradian Genes. Front Physiol. 2018;9:1178. doi:10.3389/fphys.2018.01178. 30190679 PMC6115496

[22] Zhu B. Decoding the function and regulation of the mammalian 12h-clock. Journal of Molecular Cell Biology. 2020. doi:10.1093/jmcb/mjaa021. PMC7816679 32384155

[23] Sorek M, Schnytzer Y, Waldman Ben-Asher H, Caspi VC, Chen C-S, Miller DJ, Setting the pace: host rhythmic behaviour and gene expression patterns in the facultatively symbiotic cnidarian Aiptasia are determined largely by Symbiodinium. Microbiome. 2018;6(1):83. doi:10.1186/s40168-018-0465-9. 29739445 PMC5941691

[24] Putnam NH, Srivastava M, Hellsten U, Dirks B, Chapman J, Salamov A, Sea anemone genome reveals ancestral eumetazoan gene repertoire and genomic organization. Science. 2007;317(5834):86–94. doi:10.1126/science.1139158. 17615350

[25] Sorek M, Schnytzer Y, Ben-Asher HW, Caspi VC, Chen CS, Miller DJ, Setting the pace: host rhythmic behaviour and gene expression patterns in the facultatively symbiotic cnidarian Aiptasia are determined largely by Symbiodinium. Microbiome. 2018;6(1):83. Epub 2018/05/10. doi:10.1186/s40168-018-0465-9. 29739445 PMC5941691

[26] Hewitt EW. The MHC class I antigen presentation pathway: strategies for viral immune evasion. Immunology. 2003;110(2):163–9. doi:10.1046/j.1365-2567.2003.01738.x. 14511229 PMC1783040

[27] Garrido F, Aptsiauri N, Doorduijn EM, Garcia Lora AM, van Hall T. The urgent need to recover MHC class I in cancers for effective immunotherapy. Curr Opin Immunol. 2016;39:44–51. Epub 20160118. doi:10.1016/j.coi.2015.12.007. 26796069 PMC5138279

[28] Guttman O, Le Thomas A, Marsters S, Lawrence DA, Gutgesell L, Zuazo-Gaztelu I, Antigen-derived peptides engage the ER stress sensor IRE1α to curb dendritic cell cross-presentation. J Cell Biol. 2022;221(6). Epub 2022/04/22. doi:10.1083/jcb.202111068. PMC9036094 35446348

[29] Cubillos-Ruiz JR, Silberman PC, Rutkowski MR, Chopra S, Perales-Puchalt A, Song M, ER Stress Sensor XBP1 Controls Anti-tumor Immunity by Disrupting Dendritic Cell Homeostasis. Cell. 2015;161(7):1527–38. Epub 2015/06/16. doi:10.1016/j.cell.2015.05.025. 26073941 PMC4580135

[30] Talamanca L, Gobet C, Naef F. Sex-dimorphic and age-dependent organization of 24-hour gene expression rhythms in humans. Science. 2023;379(6631):478–83. doi:doi:10.1126/science.add0846. 36730411

[31] Scott MR, Zong W, Ketchesin KD, Seney ML, Tseng GC, Zhu B, Twelve-hour rhythms in transcript expression within the human dorsolateral prefrontal cortex are altered in schizophrenia. PLOS Biology. 2023;21(1):e3001688. doi:10.1371/journal.pbio.3001688. 36693045 PMC9873190

[32] Bolger AM, Lohse M, Usadel B. Trimmomatic: a flexible trimmer for Illumina sequence data. Bioinformatics. 2014;30(15):2114–20. doi:10.1093/bioinformatics/btu170. 24695404 PMC4103590

[33] Dobin A, Davis CA, Schlesinger F, Drenkow J, Zaleski C, Jha S, STAR: ultrafast universal RNA-seq aligner. Bioinformatics. 2013;29(1):15–21. Epub 20121025. doi:10.1093/bioinformatics/bts635. 23104886 PMC3530905

[34] Trapnell C, Roberts A, Goff L, Pertea G, Kim D, Kelley DR, Differential gene and transcript expression analysis of RNA-seq experiments with TopHat and Cufflinks. Nature Protocols. 2012;7(3):562–78. doi:10.1038/nprot.2012.016. 22383036 PMC3334321

[35] Huang da W, Sherman BT, Lempicki RA. Systematic and integrative analysis of large gene lists using DAVID bioinformatics resources. Nat Protoc. 2009;4(1):44–57. doi:10.1038/nprot.2008.211. 19131956

