# Supplementary material for: Evidence for conservation of primordial ~12-hour ultradian gene programs in humans under free-living conditions
Source: bioRxiv. 2023 Dec 23:2023.05.02.539021. Originally published 2023 May 2. Preprint. [Version 2] doi: 10.1101/2023.05.02.539021 (PMC10187241; doi:10.1101/2023.05.02.539021)
Supplement: Supplement 1 [file NIHPP2023.05.02.539021V2-supplement-1.pdf]

bioRxiv preprint doi: https://doi.org/10.1101/2023.05.02.539021; this version posted December 23, 2023. The copyright holder for this preprint (which was not certified by peer review) is the author/funder, who has granted bioRxiv a license to display the preprint in perpetuity. It is made available under aCC-BY 4.0 International license.

Supplementary Materials for

Conservation of a primordial 12-hour ultradian gene program in humans

Bokai Zhu[1,2,3] *, Silvia Liu[2,4], Natalie L. David[1,5,6], William Dion[1], Nandini K Doshi[1,6], Lauren B. Siegel[5], Tânia Amorim[1,5,6], Rosemary E. Andrews[1,6], GV Naveen Kumar[1], Hanwen Li[7], Saad Irfan[1], Tristan Pesaresi[1,6], Ankit X. Sharma[1], Michelle Sun[1], Pouneh K. Fazeli [5,6] and Matthew L. Steinhauser [1,6,8] *

Corresponding author: bzhu@pitt.edu, msteinhauser@pitt.edu

bioRxiv preprint doi: https://doi.org/10.1101/2023.05.02.539021; this version posted December 23, 2023. The copyright holder for this preprint (which was not certified by peer review) is the author/funder, who has granted bioRxiv a license to display the preprint in perpetuity. It is made available under aCC-BY 4.0 International license.

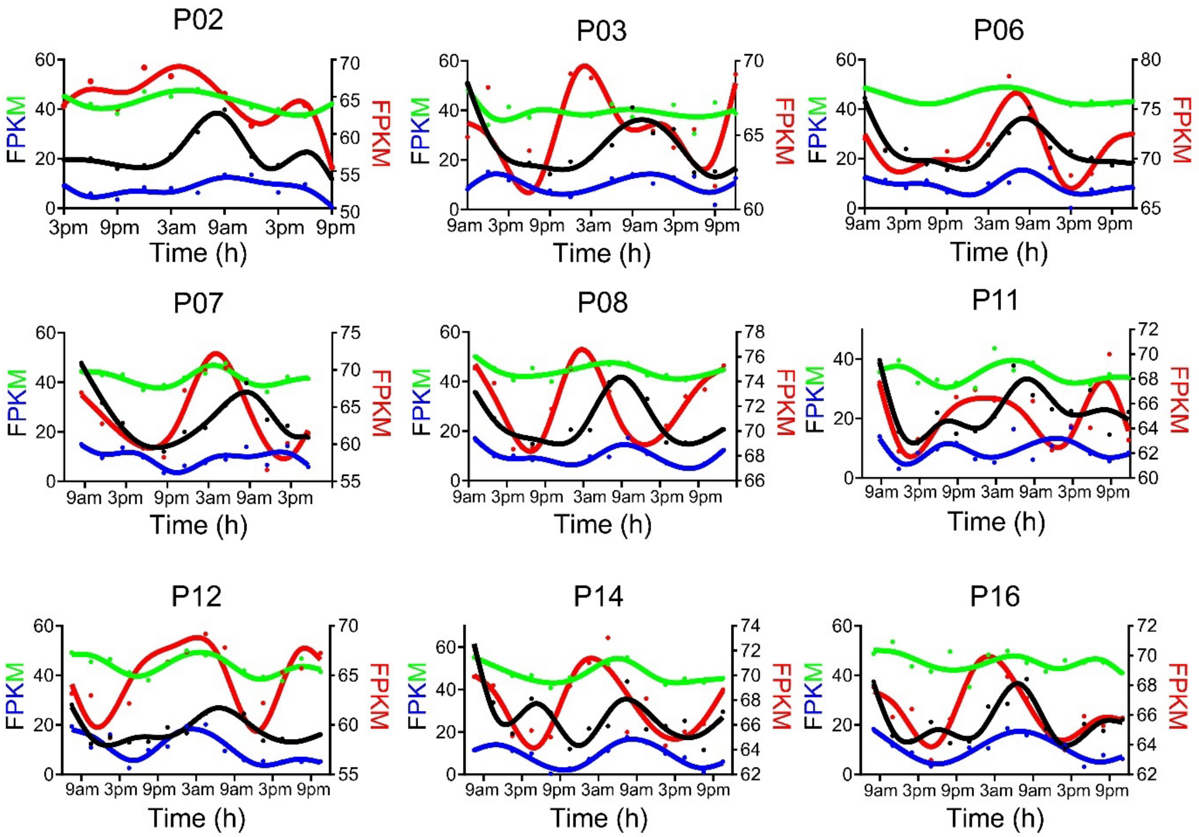

Fig. S1. Inter-individual variability of circadian rhythms of gene expression reported in the Wittenbrink et al JCI study. Raw temporal expression profile (dot) and spline fit (solid line) of *PER1* (black), *PER2* (blue), *TSPAN4* (green) and *LGALS3* (red) genes in nine different individuals, reported in [14].

bioRxiv preprint doi: https://doi.org/10.1101/2023.05.02.539021; this version posted December 23, 2023. The copyright holder for this preprint (which was not certified by peer review) is the author/funder, who has granted bioRxiv a license to display the preprint in perpetuity. It is made available under aCC-BY 4.0 International license.

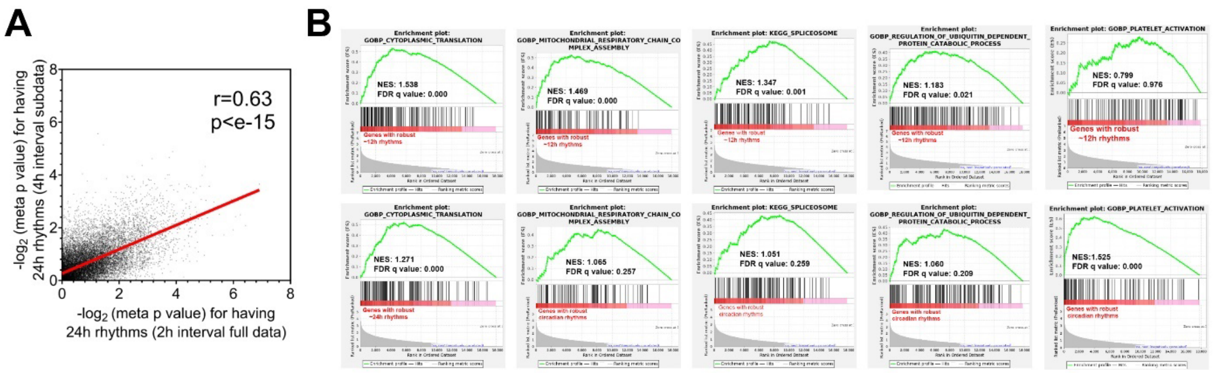

Fig. S2. Genes with ~12h and circadian rhythms are enriched in distinct biological pathways. (**A**) Scatter plot comparing log normalized meta p values for having circadian rhythms for each gene when the full 2h sampling interval dataset (x-axis) or the 4h sampling interval subset (y-axis) was used for the analysis. (**B**) GSEA showing enrichment scores for different gene sets in ~12h (top) and circadian (bottom) genes.

bioRxiv preprint doi: https://doi.org/10.1101/2023.05.02.539021; this version posted December 23, 2023. The copyright holder for this preprint (which was not certified by peer review) is the author/funder, who has granted bioRxiv a license to display the preprint in perpetuity. It is made available under aCC-BY 4.0 International license.

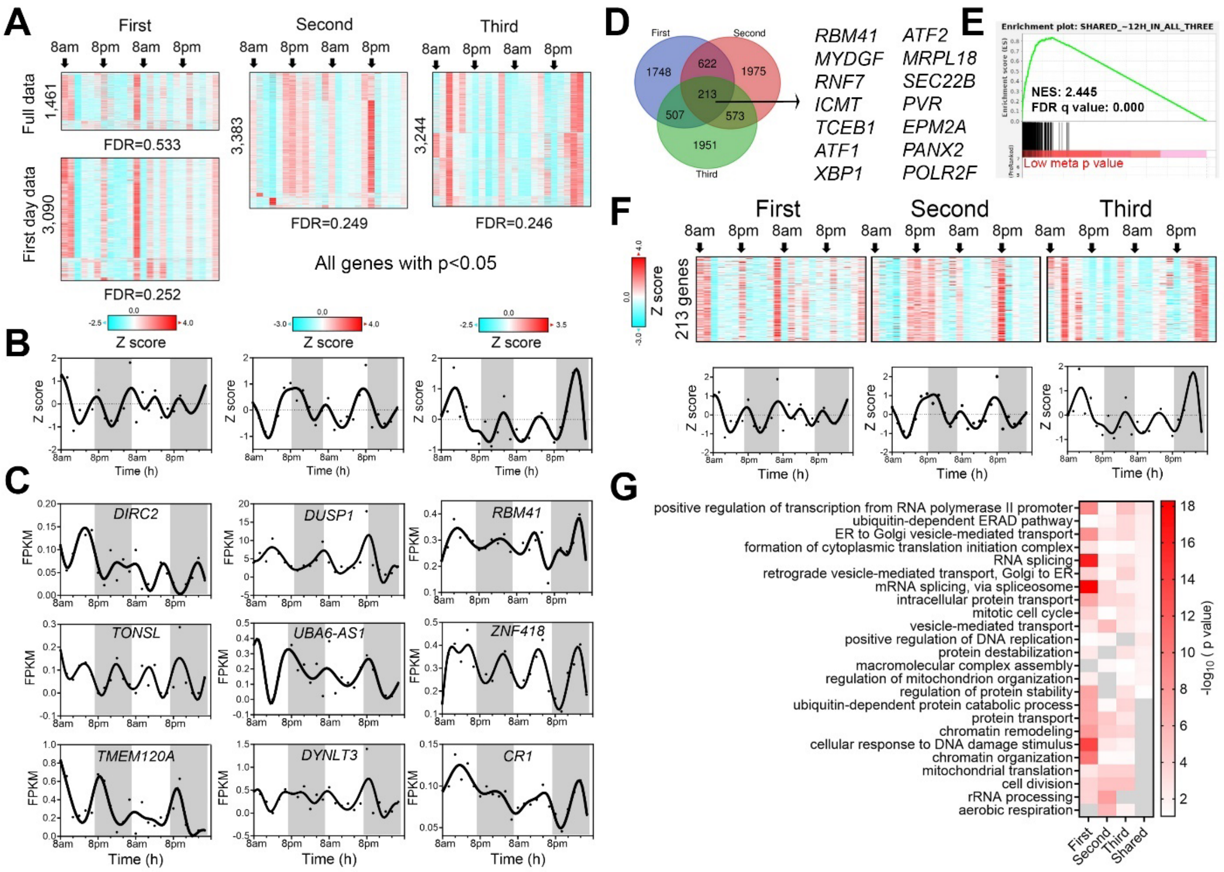

Fig. S3. RAIN analyses of 48h temporal transcriptome to detect ~12h genes. A single data point was inputted for each sample collected over the 48h protocol. (**A**) Heatmap of ~12h genes from all three participants with respective p values smaller than 0.05. The identification of a greater number of ~12h genes with lower FDR in the first participant with restriction of inputted data to the first 24 hours indicates the ~12h rhythm is dampened in the second day in this participant, consistent with what was found with the eigenvalue/pencil method. (**B**) Quantification of the average expression (Z score normalized) of genes shown in A, with raw data (dots) and spline fit (solid lines) shown. (**C**) Raw temporal expression (dot) profile and spline fit (solid line) of top three ~12h genes with the smallest p values in each of the three individuals. (**D**) Venn diagram depicting common and distinct ~12h genes for each individual, with selective common genes shown on the right. (**E**) GSEA showing enrichment score of 213 common ~12h genes on robust ~12h genes ranked by meta p values. (**F**) Heatmap and quantification of 213 common ~12h genes uncovered from all three participants. (**G**) GO analysis of all and shared ~12h genes in each individual.

bioRxiv preprint doi: https://doi.org/10.1101/2023.05.02.539021; this version posted December 23, 2023. The copyright holder for this preprint (which was not certified by peer review) is the author/funder, who has granted bioRxiv a license to display the preprint in perpetuity. It is made available under aCC-BY 4.0 International license.

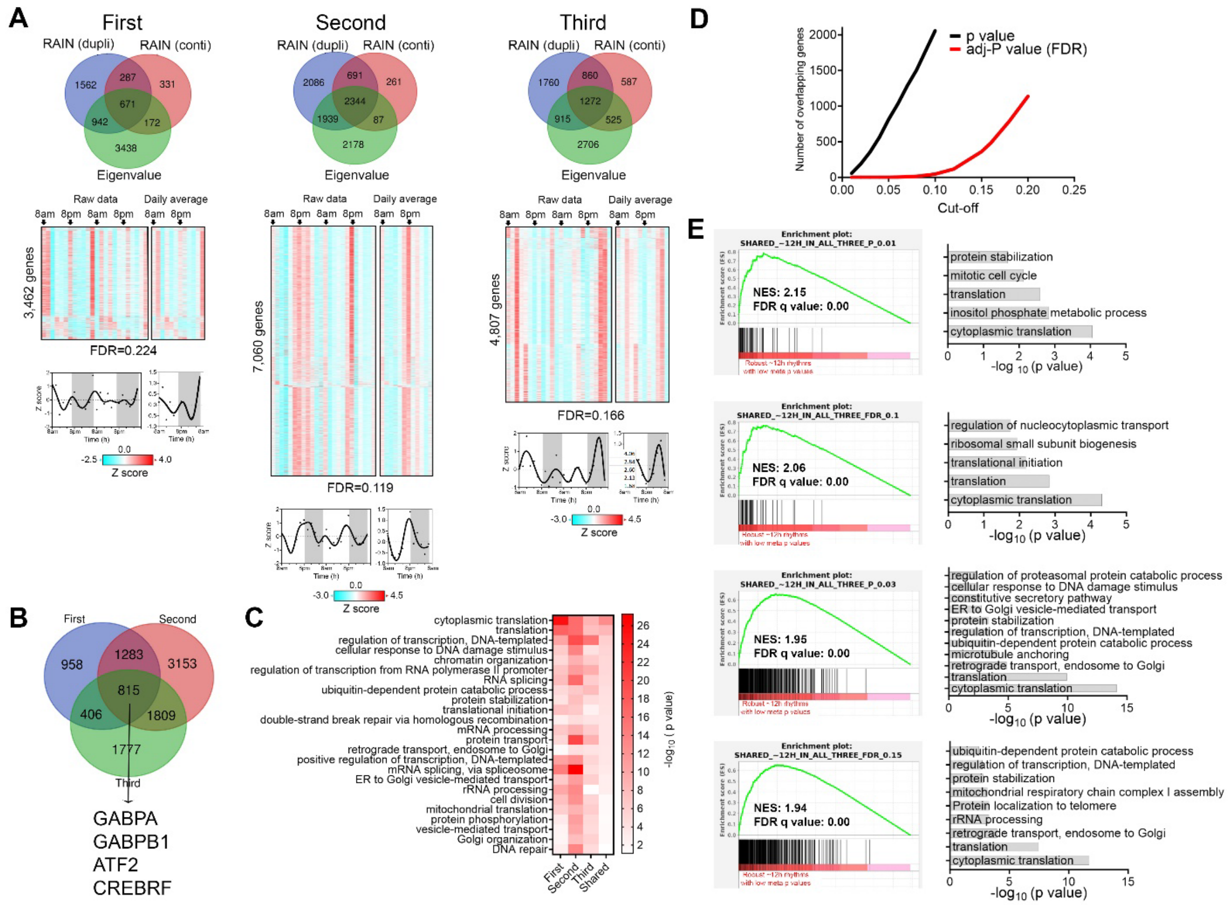

Fig. S4. RAIN analyses of temporal transcriptomes using consecutive 24 hours datasets as biological replicates. (**A**) Venn diagram comparing the ~12h programs uncovered by the three methods (top) and heatmap and quantification of ~12h genes uncovered by the RAIN dupli method in all three participants (bottom). (**B**) Venn diagram depicting common and distinct ~12h genes uncovered in each participant. (**C**) GO analysis of all and shared ~12h genes in all three participants. (**D**) Scatter plot comparing the number of overlapping ~12h genes between the three individuals against different p values or FDR cut-offs for all individuals. (**E**) GSEA showing enrichment scores for four gene sets of overlapping ~12h genes with p<0.01 (56 genes), p<0.03 (358 genes), FDR<0.1 (46 genes) and FDR<0.15 (361 genes) cut-off (left) on ~12h genes ranked by meta-p values and GO analysis of these genes (right).

bioRxiv preprint doi: https://doi.org/10.1101/2023.05.02.539021; this version posted December 23, 2023. The copyright holder for this preprint (which was not certified by peer review) is the author/funder, who has granted bioRxiv a license to display the preprint in perpetuity. It is made available under aCC-BY 4.0 International license.

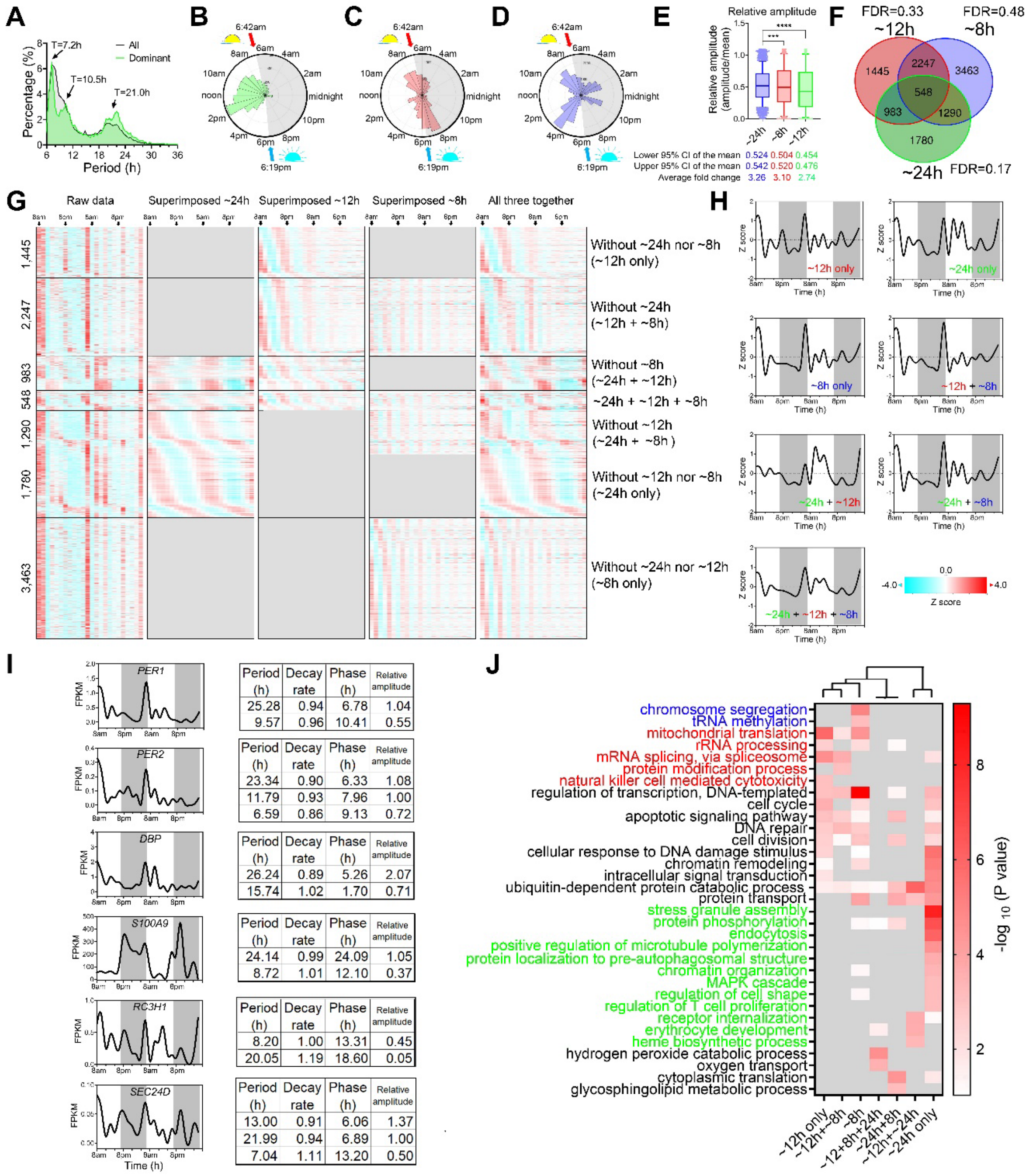

Fig. S5. Transcriptome spectrum of first human participant. (**A**) Period distribution of all and dominant oscillations. (**B-D**) Polar histogram illustrating the phase distribution of ~24h (**B**), ~12h (**C**) and ~8h (**D**) oscillations. (**E**) Relative amplitude (mean-normalized) of ~8h, ~12h and ~24h oscillations. (**F**) Venn diagram showing distinct and shared oscillations for each period. (**G, H**) Heatmap (**G**) and quantification (**H**) of decomposition of raw temporal transcriptome into harmonics cycling at ~8h, ~12h or ~24h periods. (**I**) Representative temporal expression of select genes and eigenvalue/pencil decomposition uncovering all superimposed oscillations for each gene. (**J**) GO analysis of all genes in **G**.

bioRxiv preprint doi: https://doi.org/10.1101/2023.05.02.539021; this version posted December 23, 2023. The copyright holder for this preprint (which was not certified by peer review) is the author/funder, who has granted bioRxiv a license to display the preprint in perpetuity. It is made available under aCC-BY 4.0 International license.

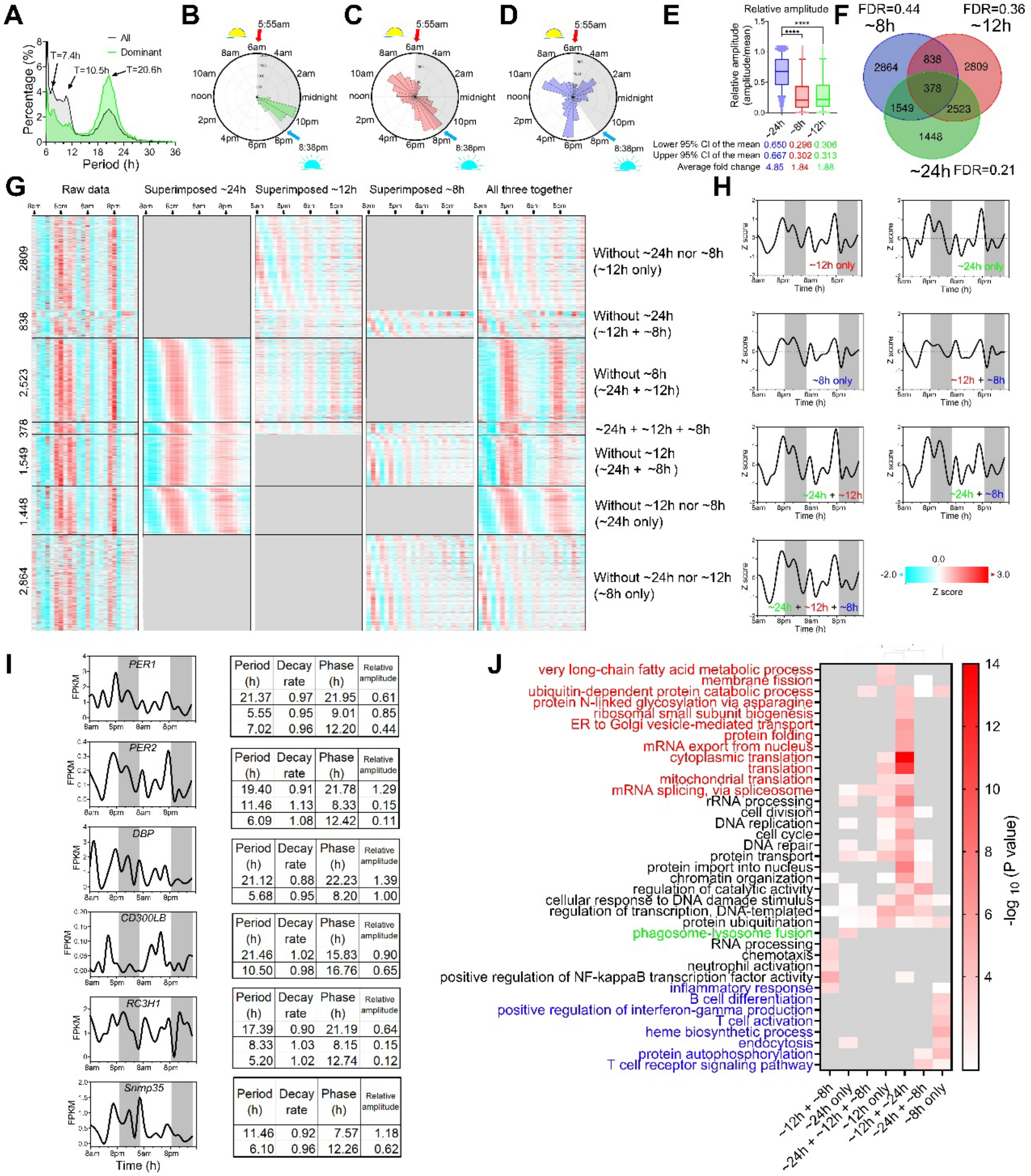

Fig. S6. **Transcriptome spectrum of the second human participant.** (**A**) Period distribution of all and dominant oscillations. (**B-D**) Polar histogram illustrating the phase distribution of ~24h (**B**), ~12h (**C**) and ~8h (**D**) oscillations. (**E**) Relative amplitude (mean-normalized) of ~8h, ~12h and ~24h oscillations. (**F**) Venn diagram showing distinct and shared oscillations for each period. (**G, H**) Heatmap (**G**) and quantification (**H**) of decomposition of raw temporal transcriptome into harmonics cycling at ~8h, ~12h or ~24h periods. (**I**) Representative temporal expression of selective genes and eigenvalue/pencil decomposition uncovering all superimposed oscillations for each gene. (**J**) GO analysis of all genes in **G**.

bioRxiv preprint doi: https://doi.org/10.1101/2023.05.02.539021; this version posted December 23, 2023. The copyright holder for this preprint (which was not certified by peer review) is the author/funder, who has granted bioRxiv a license to display the preprint in perpetuity. It is made available under aCC-BY 4.0 International license.

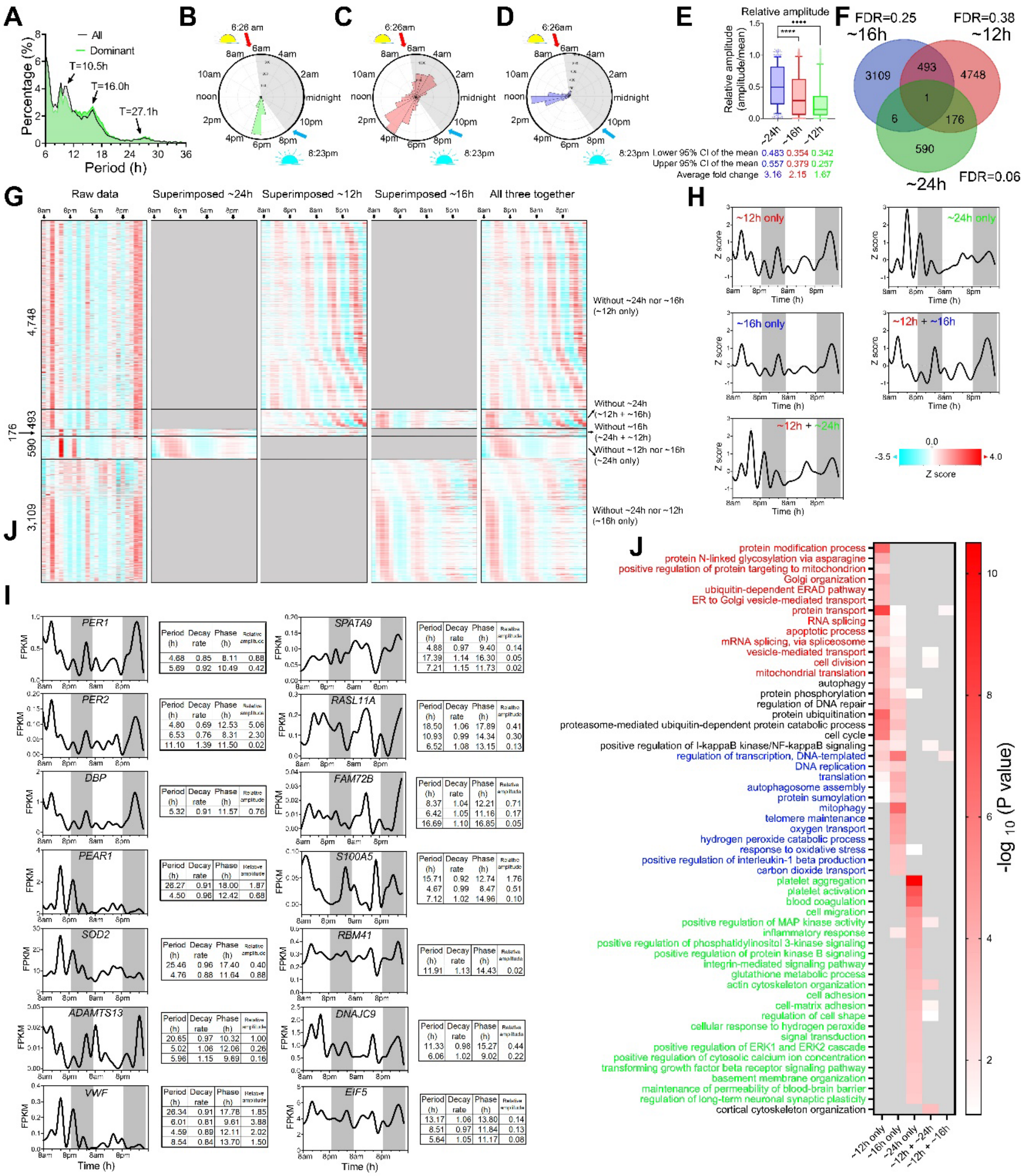

Fig. S7. Transcriptome spectrum of the third human participant. (**A**) Period distribution of all and dominant oscillations. (**B-D**) Polar histogram illustrating the phase distribution of ~24h (**B**), ~12h (**C**) and ~16h (**D**) oscillations. (**E**) Relative amplitude (mean-normalized) of ~16h, ~12h and ~24h oscillations. (**F**) Venn diagram showing distinct and shared oscillations for each period. (**G, H**) Heatmap (**G**) and quantification (**H**) of decomposition of raw temporal transcriptome into oscillations cycling at ~16h, ~12h or ~24h periods. (**I**) Representative temporal expression of selective genes and eigenvalue/pencil decomposition uncovering all superimposed oscillations for each gene. (**J**) GO analysis of all genes in **G**.

bioRxiv preprint doi: https://doi.org/10.1101/2023.05.02.539021; this version posted December 23, 2023. The copyright holder for this preprint (which was not certified by peer review) is the author/funder, who has granted bioRxiv a license to display the preprint in perpetuity. It is made available under aCC-BY 4.0 International license.

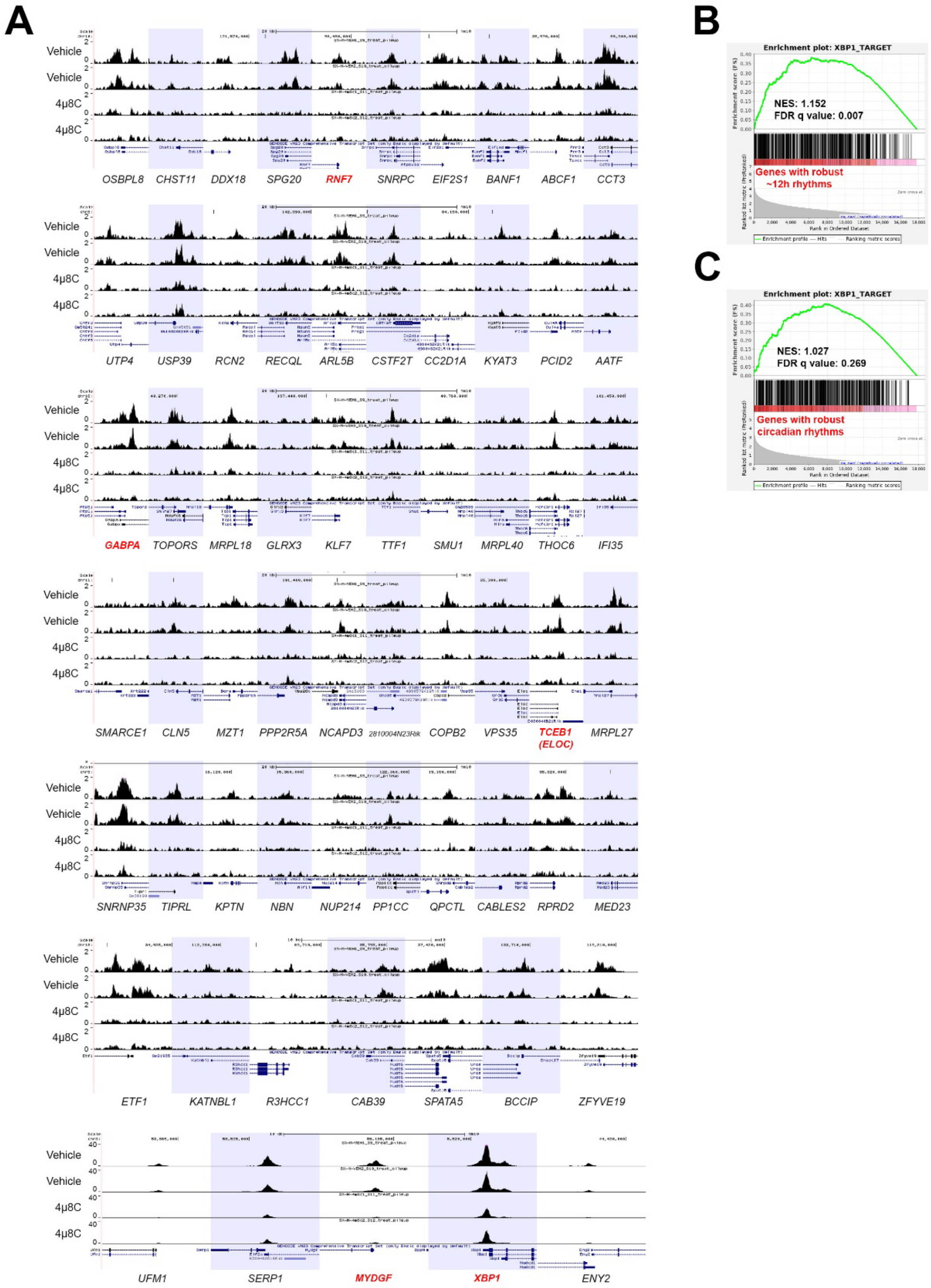

Fig. S8. XBP1 chromatin binding landscape of 12h genes in mouse Th2 cells. (**A**) UCSC genome browser view of XBP1 chromatin recruitment to the promoters of a selected set of ~12h genes in murine Th2 cells. (**B, C**) GSEA showing enrichment score of top 500 XBP1 target genes in Th2 cells (ranked by fold reduction of target gene expression with XBP1 deletion) in robust ~12h (**B**) or circadian (**C**) genes identified in humans.

bioRxiv preprint doi: https://doi.org/10.1101/2023.05.02.539021; this version posted December 23, 2023. The copyright holder for this preprint (which was not certified by peer review) is the author/funder, who has granted bioRxiv a license to display the preprint in perpetuity. It is made available under aCC-BY 4.0 International license.

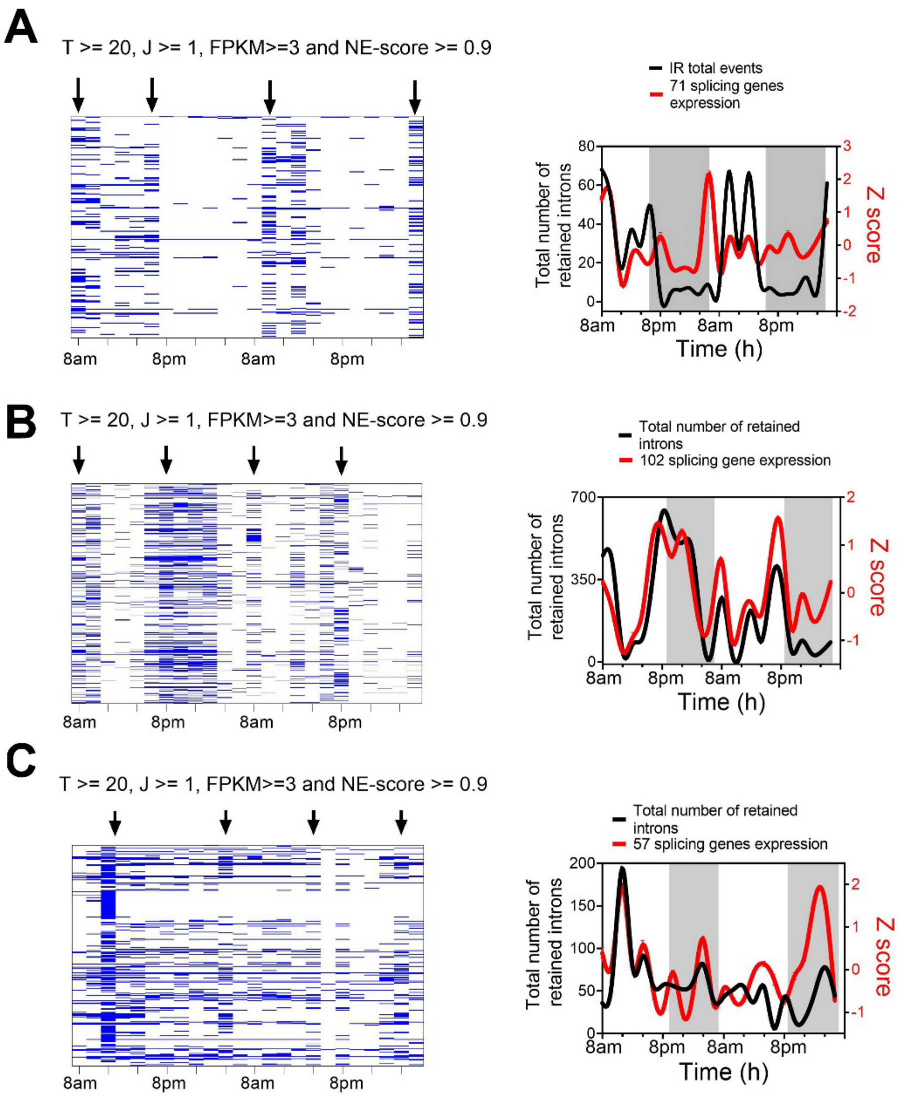

D

	Intron retention events rhythms	
	Period	p value
First	10h	0.2041
Second	12h	0.0439
Third	12h	0.0056
Meta	10-12h	2.990E-03

Fig. S9. ~12h rhythms are synchronized to global intron retention rhythms. Criterions for IR are set as T>=20, J>=1, FPKM>=3 and NE score>=0.9. (**A-C**) Heatmap (left) and quantification (right) of temporal IR events, superimposed with the Z score normalized temporal expression of ~12h splicing gene expression in the first (**A**), second (**B**) and third (**C**) participants. IR events are detected with the more stringent setting of the iRead algorithm as described in the methods section. (**D**) Statistics for IR ~12h rhythms detection by RAIN.

bioRxiv preprint doi: https://doi.org/10.1101/2023.05.02.539021; this version posted December 23, 2023. The copyright holder for this preprint (which was not certified by peer review) is the author/funder, who has granted bioRxiv a license to display the preprint in perpetuity. It is made available under aCC-BY 4.0 International license.

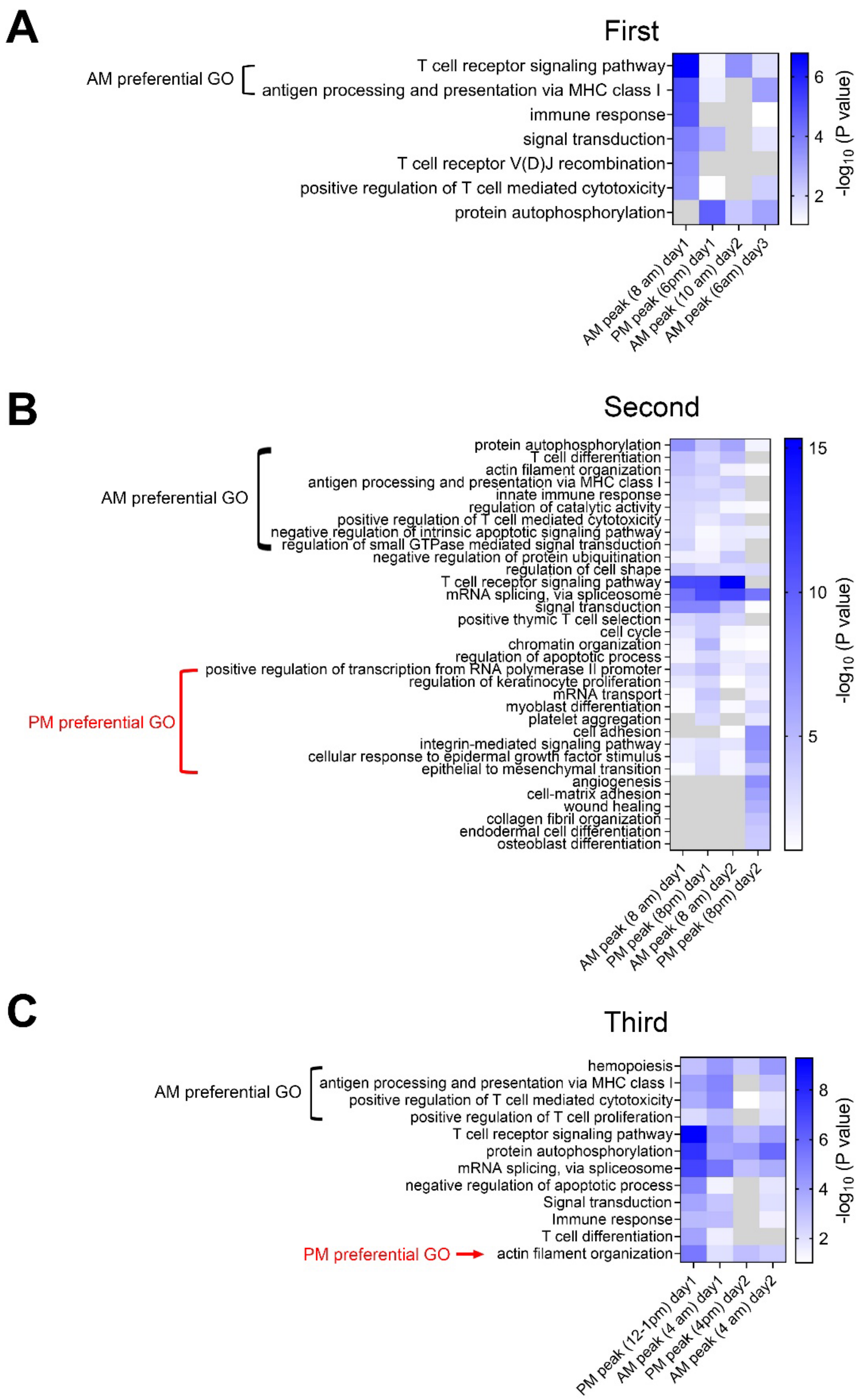

Fig. S10. Genes with intron retentions are enriched in immune genes. Intron retention events are selected with default settings T>=20, J>=1, FPKM>=2 and NE score>=0.9. GO analysis of IR genes at different peaks in the first (**A**), second (**B**) and third (**C**) participant.

bioRxiv preprint doi: https://doi.org/10.1101/2023.05.02.539021; this version posted December 23, 2023. The copyright holder for this preprint (which was not certified by peer review) is the author/funder, who has granted bioRxiv a license to display the preprint in perpetuity. It is made available under aCC-BY 4.0 International license.

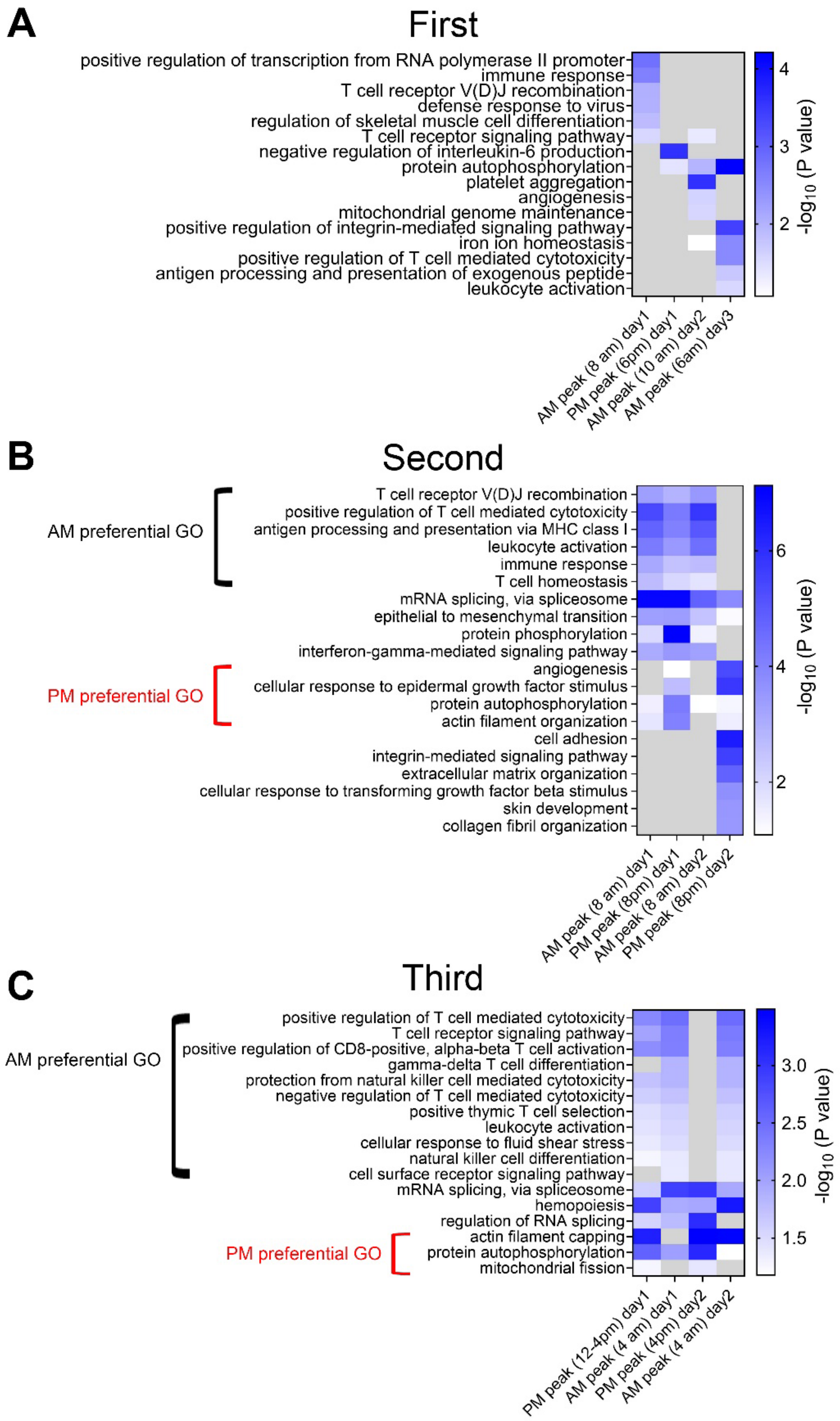

Fig. S11. Genes with intron retentions (more stringent criterion) are enriched in immune genes. Intron retention events are selected with default settings T>=20, J>=1, FPKM>=3 and NE score>=0.9. GO analysis of IR genes at different peaks in the first (**A**), second (**B**) and third (**C**) participant.

bioRxiv preprint doi: https://doi.org/10.1101/2023.05.02.539021; this version posted December 23, 2023. The copyright holder for this preprint (which was not certified by peer review) is the author/funder, who has granted bioRxiv a license to display the preprint in perpetuity. It is made available under aCC-BY 4.0 International license.

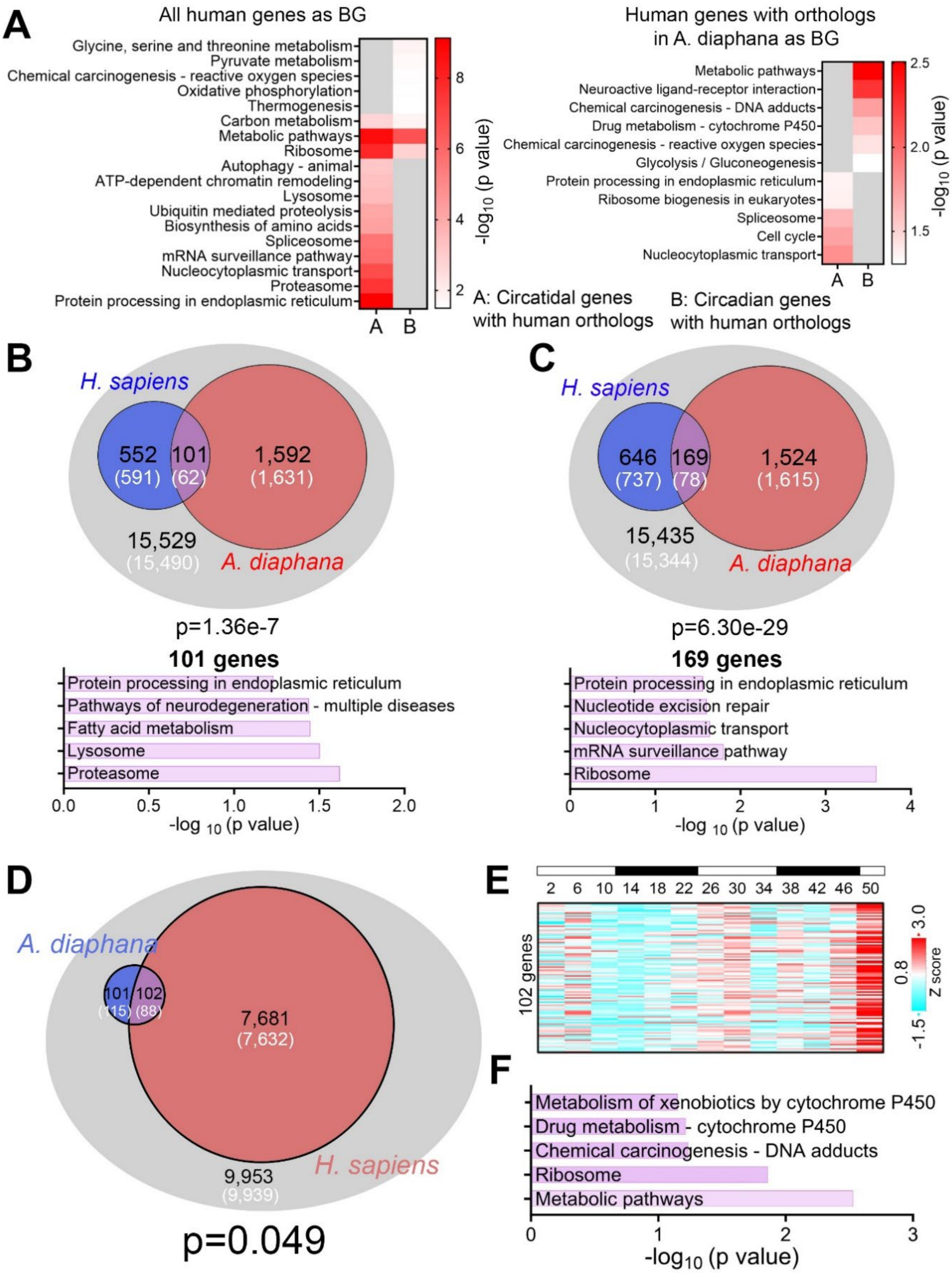

1003
1004 **Fig. S12. Evolutionary conservation of ~12h, but not circadian gene programs between A.**
1005 **diaphana and human.** (**A**) GO analysis of all circatidal genes in A. diaphana that have human
1006 orthologs using all human genes (left) or only those with A. diaphana orthologs (right) as

bioRxiv preprint doi: https://doi.org/10.1101/2023.05.02.539021; this version posted December 23, 2023. The copyright holder for this preprint (which was not certified by peer review) is the author/funder, who has granted bioRxiv a license to display the preprint in perpetuity. It is made available under aCC-BY 4.0 International license.

background. (**B, C**) Venn diagram comparing distinct and shared ~12h genes in human (653 12h genes with meta adj-P<0.05 in **B** and 851 ~12h genes reported in Fig. S4B in **C** and *A. diaphana* (reported in [44]). Only genes that are expressed in human white blood cells (denoted by the grey circle) are included in the analysis. Both observed and predicted number of genes (under the null hypothesis that ~12h genes are not evolutionarily conserved and thus independently detected in these two species) are further shown. P values are calculated by the Chi-square test. GO analysis showing enriched KEGG terms for the 101 and 169 common genes, respectively. (**D**) Venn diagram comparing distinct and shared circadian genes in human (meta adj-P<0.1) and *A. diaphana* (reported in [44]). Only genes that are expressed in human white blood cells (denoted by the grey circle) are included in the analysis. Both observed and predicted number of genes (under the null hypothesis that circadian genes are not evolutionarily conserved and thus independently detected in these two species) are further shown. P value of 0.049 is calculated by Chi-square test. (**E, F**) Heatmap of temporal expression (Z score normalized) of 102 circadian genes in *A. diaphana* (**E**) and GO analysis of the top enriched pathways (**F**).

Table S1. RPKM quantification of temporal expression for the human participants

Table S2. RAIN results for ~12h genes and meta-analysis of circadian genes.

Table S3. Eigenvalue/pencil results
