# Supplementary material for: Evidence for conservation of a primordial 12-hour ultradian gene program in humans
Source: bioRxiv. 2023 May 2:2023.05.02.539021. Preprint. [Version 1] doi: 10.1101/2023.05.02.539021 (PMC10187241; doi:10.1101/2023.05.02.539021)
Supplement: Supplementary file 1 [file NIHPP2023.05.02.539021v1-supplement-1.pdf]

bioRxiv preprint doi: https://doi.org/10.1101/2023.05.02.539021; this version posted May 2, 2023. The copyright holder for this preprint (which was not certified by peer review) is the author/funder, who has granted bioRxiv a license to display the preprint in perpetuity. It is made available under aCC-BY 4.0 International license.

Supplemental Information

Conservation of a primordial 12-hour ultradian gene program in humans

Bokai Zhu[1,2,3] *, Silvia Liu[2,4], Natalie L. David[1,5,6], William Dion[1], Nandini K Doshi[1,6], Lauren B. Siegel[5], Tânia Amorim[1,5,6], Rosemary E. Andrews[1,6], GV Naveen Kumar[1], Saad Irfan[1], Tristan Pesaresi[1,6], Ankit X. Sharma[1], Michelle Sun[1], Pouneh K. Fazeli [5,6] and Matthew L. Steinhauser [1,6,7] *

Corresponding author: bzhu@pitt.edu, msteinhauser@pitt.edu

bioRxiv preprint doi: https://doi.org/10.1101/2023.05.02.539021; this version posted May 2, 2023. The copyright holder for this preprint (which was not certified by peer review) is the author/funder, who has granted bioRxiv a license to display the preprint in perpetuity. It is made available under aCC-BY 4.0 International license.

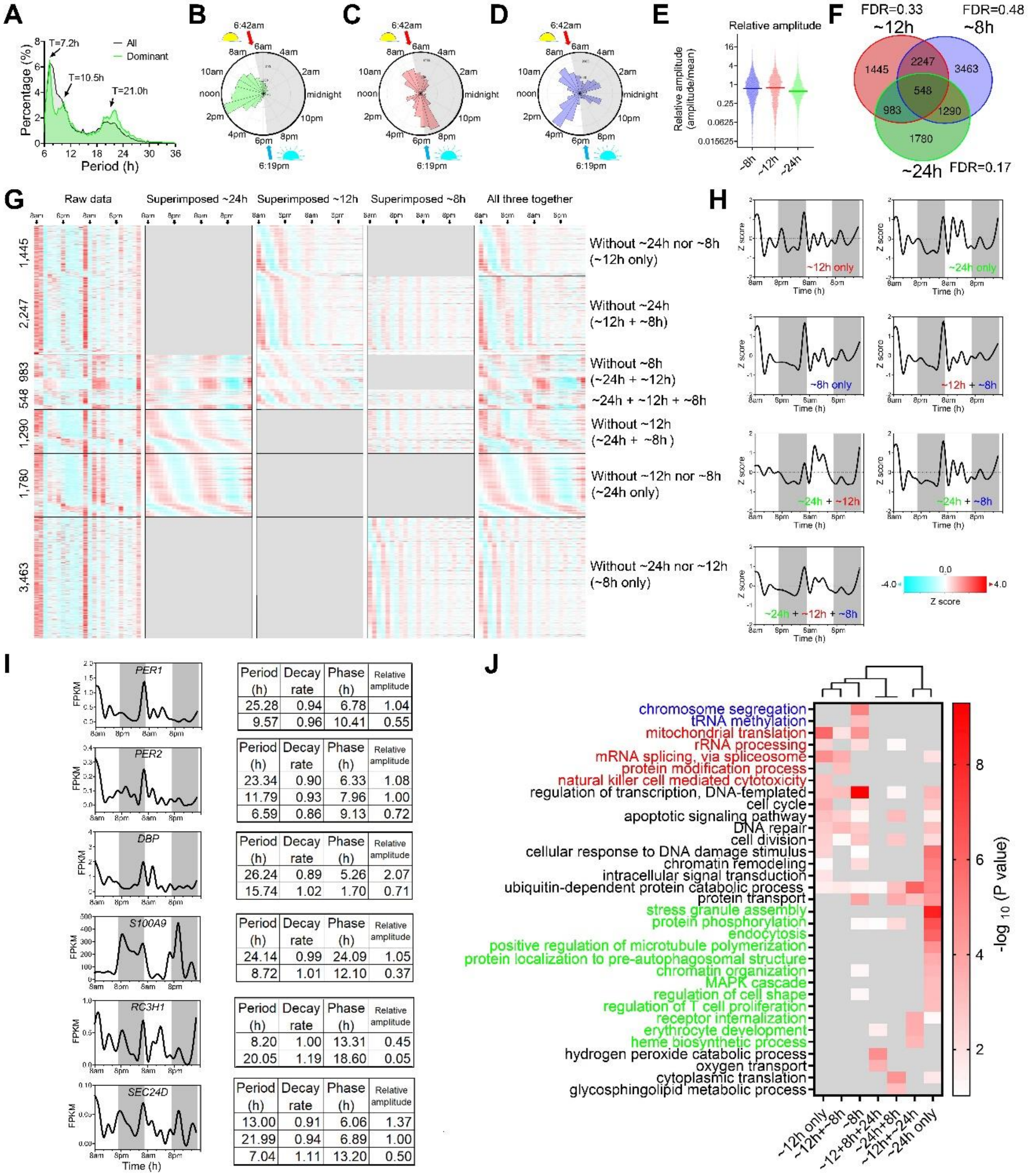

Fig. S1. Transcriptome spectrum of the first human subject. (**A**) Period distribution of all and dominant oscillations. (**B-D**) Polar histogram illustrating the phase distribution of ~24h (**B**), ~12h (**C**) and ~8h (**D**) oscillations. (**E**) Relative amplitude (mean-normalized) of ~8h, ~12h and ~24h oscillations. (**F**) Venn diagram showing distinct and shared oscillations for each period. (**G, H**) Heatmap (**G**) and quantification (**H**) of decomposition of raw temporal transcriptome into harmonics cycling at ~8h, ~12h or ~24h periods. (**I**) Representative temporal expression of select genes and eigenvalue/pencil decomposition uncovering all superimposed oscillations for each gene. (**J**) GO analysis of all genes in **G**.

bioRxiv preprint doi: https://doi.org/10.1101/2023.05.02.539021; this version posted May 2, 2023. The copyright holder for this preprint (which was not certified by peer review) is the author/funder, who has granted bioRxiv a license to display the preprint in perpetuity. It is made available under aCC-BY 4.0 International license.

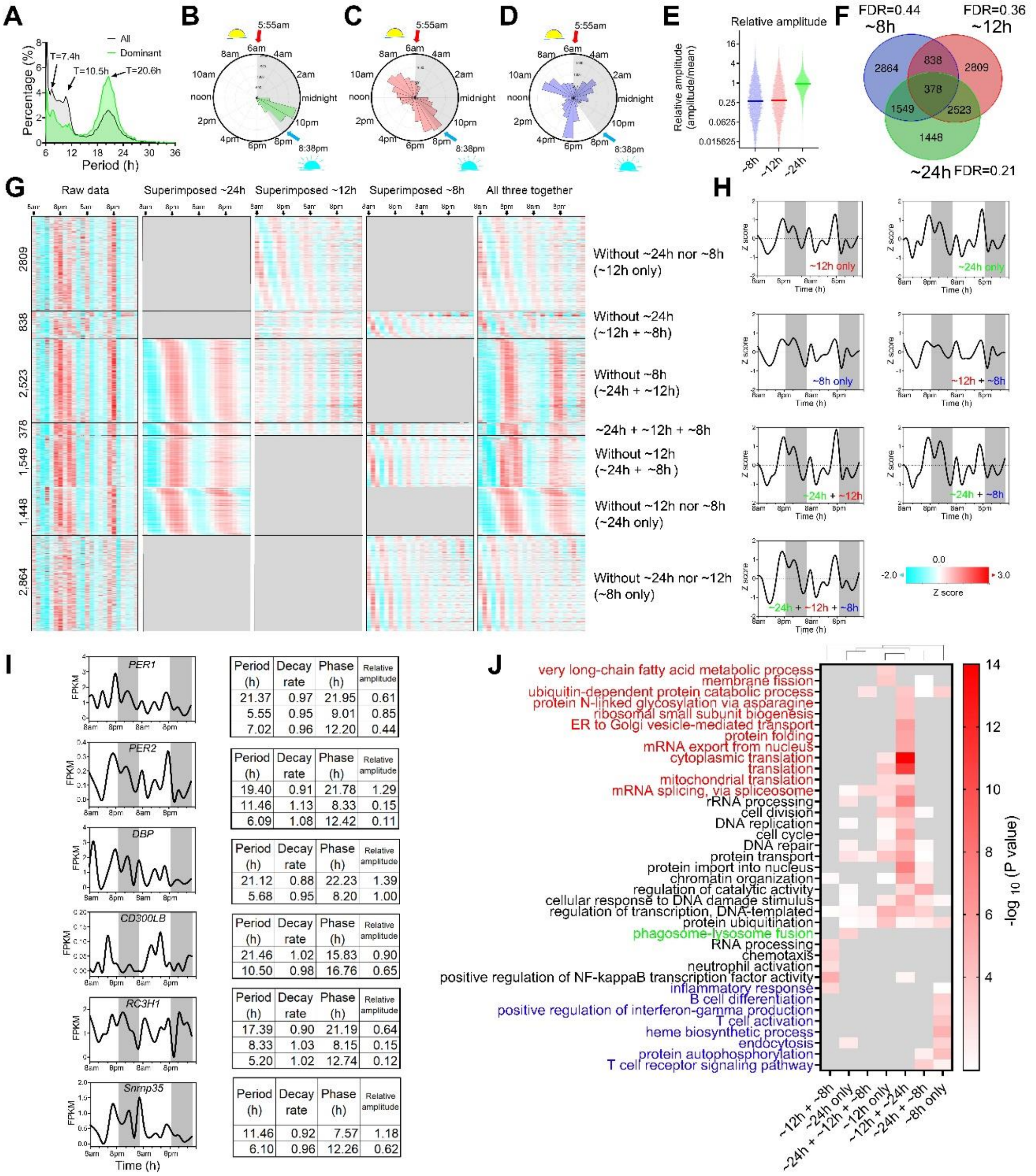

Fig. S2. Transcriptome spectrum of the second human subject. (**A**) Period distribution of all and dominant oscillations. (**B-D**) Polar histogram illustrating the phase distribution of ~24h (**B**), ~12h (**C**) and ~8h (**D**) oscillations. (**E**) Relative amplitude (mean-normalized) of ~8h, ~12h and ~24h oscillations. (**F**) Venn diagram showing distinct and shared oscillations for each period. (**G, H**) Heatmap (**G**) and quantification (**H**) of decomposition of raw temporal transcriptome into harmonics cycling at ~8h, ~12h or ~24h periods. (**I**) Representative temporal expression of selective genes and eigenvalue/pencil decomposition uncovering all superimposed oscillations for each gene. (**J**) GO analysis of all genes in **G**.

bioRxiv preprint doi: https://doi.org/10.1101/2023.05.02.539021; this version posted May 2, 2023. The copyright holder for this preprint (which was not certified by peer review) is the author/funder, who has granted bioRxiv a license to display the preprint in perpetuity. It is made available under aCC-BY 4.0 International license.

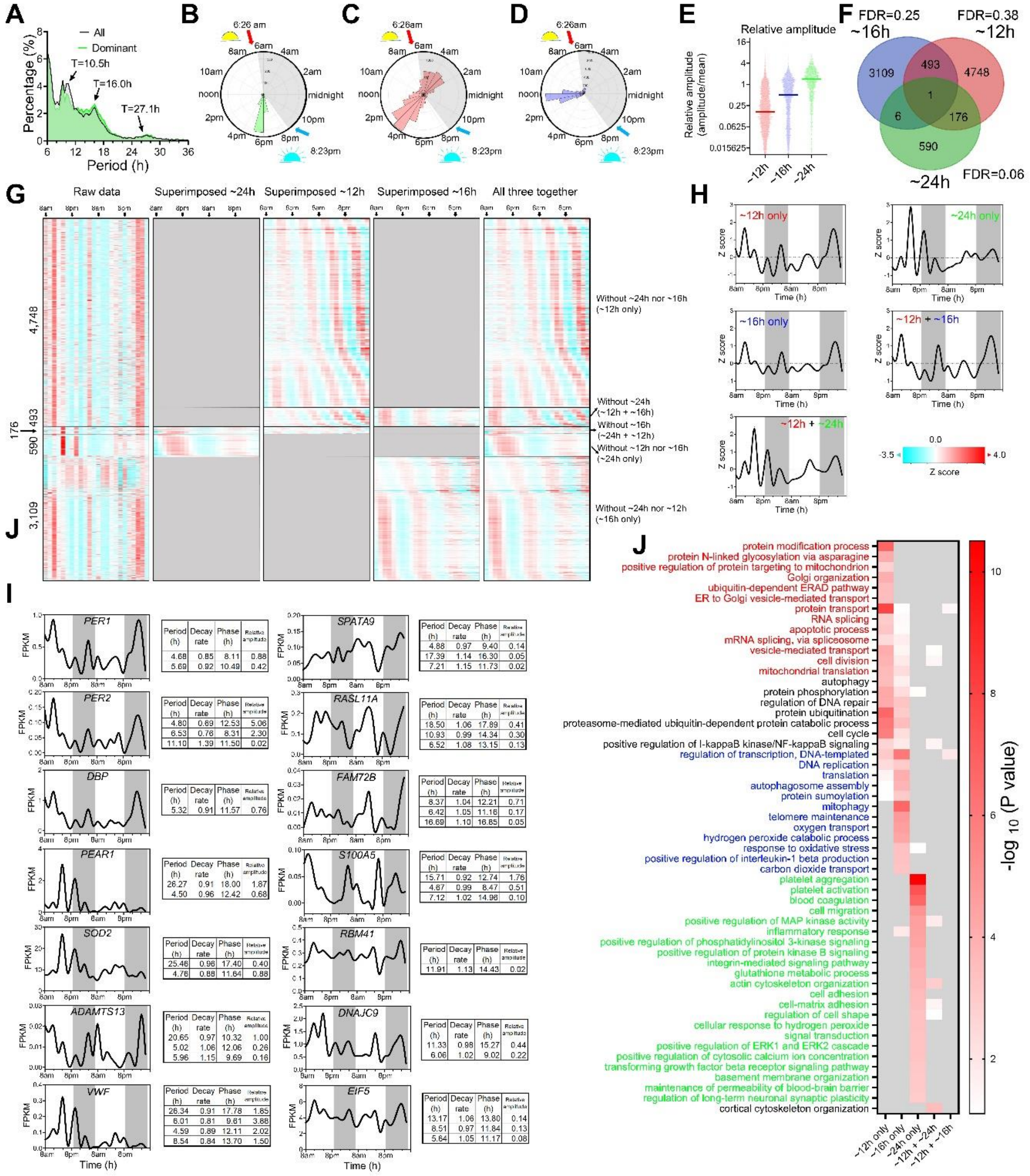

Fig. S3. Transcriptome spectrum of the third human subject. (**A**) Period distribution of all and dominant oscillations. (**B-D**) Polar histogram illustrating the phase distribution of ~24h (**B**), ~12h (**C**) and ~16h (**D**) oscillations. (**E**) Relative amplitude (mean-normalized) of ~16h, ~12h and ~24h oscillations. (**F**) Venn diagram showing distinct and shared oscillations for each period. (**G, H**) Heatmap (**G**) and quantification (**H**) of decomposition of raw temporal transcriptome into oscillations cycling at ~16h, ~12h or ~24h periods. (**I**) Representative temporal expression of selective genes and eigenvalue/pencil decomposition uncovering all superimposed oscillations for each gene. (**J**) GO analysis of all genes in **G**.

bioRxiv preprint doi: https://doi.org/10.1101/2023.05.02.539021; this version posted May 2, 2023. The copyright holder for this preprint (which was not certified by peer review) is the author/funder, who has granted bioRxiv a license to display the preprint in perpetuity. It is made available under aCC-BY 4.0 International license.

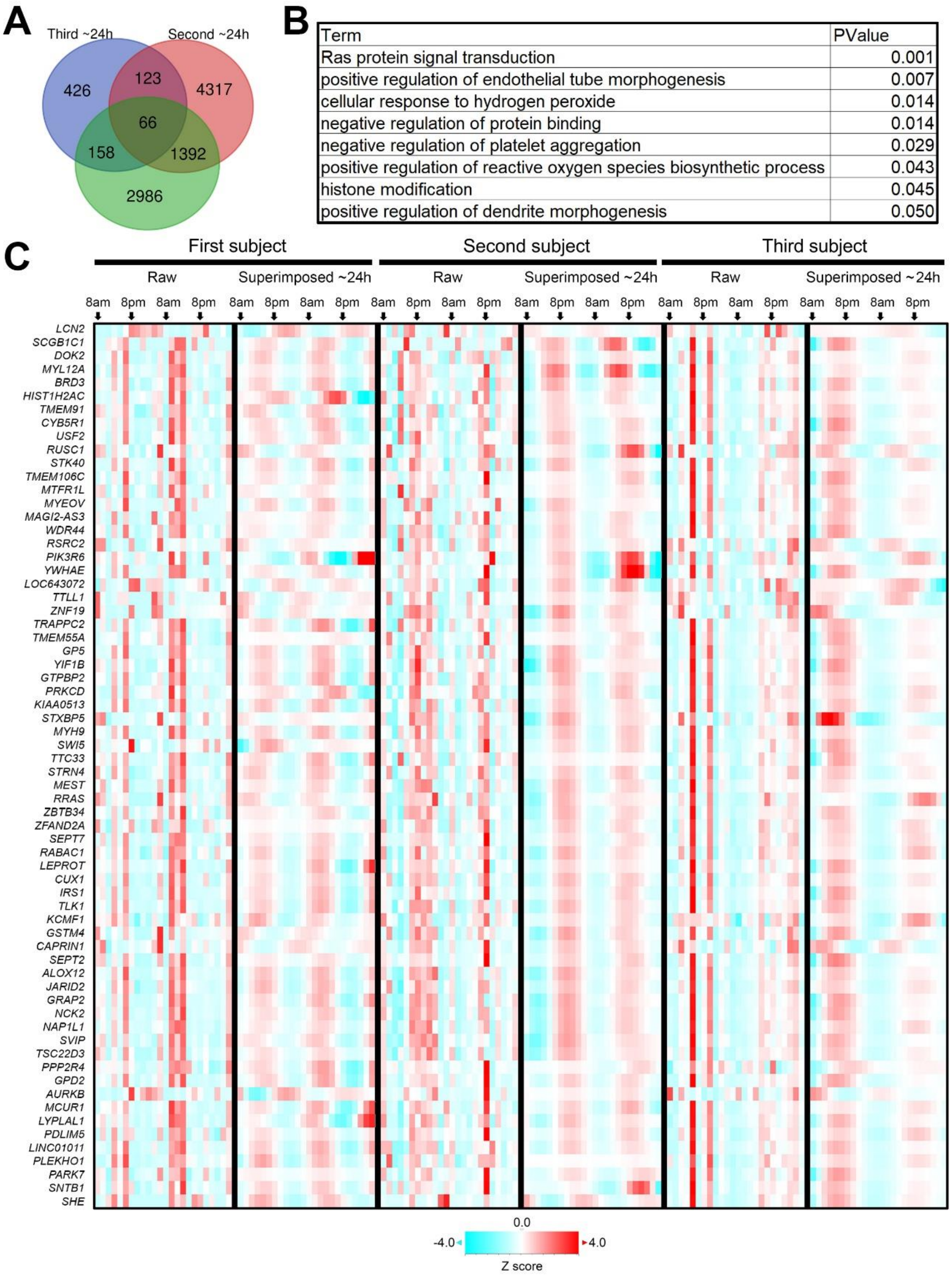

Term	PValue
Ras protein signal transduction	0.001
positive regulation of endothelial tube morphogenesis	0.007
cellular response to hydrogen peroxide	0.014
negative regulation of protein binding	0.014
negative regulation of platelet aggregation	0.029
positive regulation of reactive oxygen species biosynthetic process	0.043
histone modification	0.045
positive regulation of dendrite morphogenesis	0.050

Fig. S4. Shared circadian genes among the three subjects. (**A**) Venn diagram showing shared and distinct circadian genes in the three subjects. (**B**) GO analysis of shared circadian genes. (**C**) Heatmap showing raw as well as superimposed circadian oscillation.

bioRxiv preprint doi: https://doi.org/10.1101/2023.05.02.539021; this version posted May 2, 2023. The copyright holder for this preprint (which was not certified by peer review) is the author/funder, who has granted bioRxiv a license to display the preprint in perpetuity. It is made available under aCC-BY 4.0 International license.

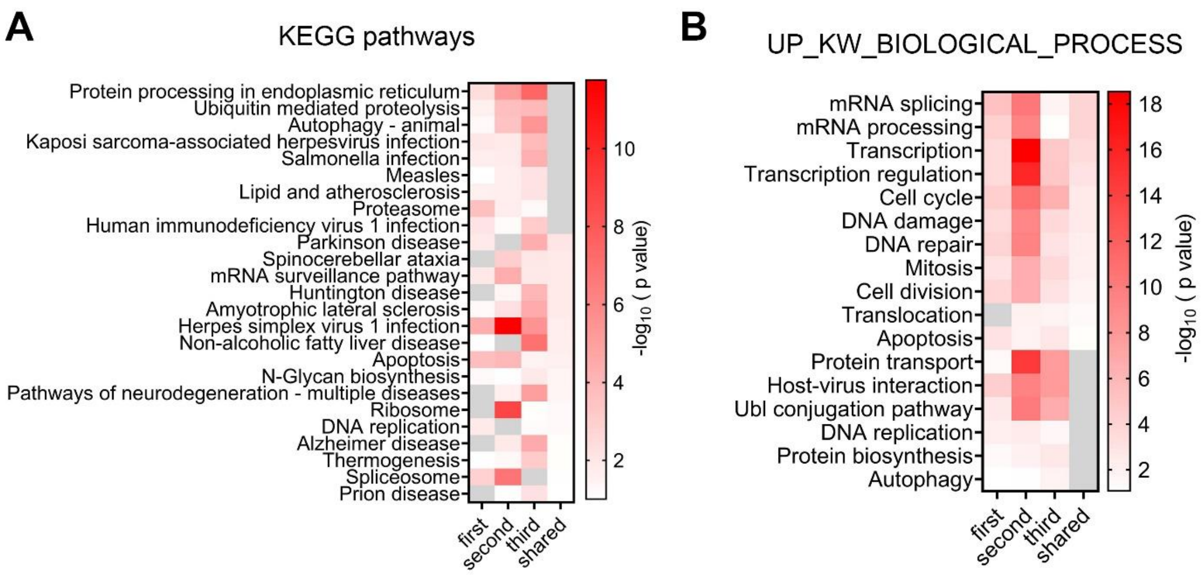

Fig. S5. GO analysis of the ~12h rhythm transcriptome. GO analysis of all and shared ~12h gene transcripts using KEGG (**A**) or UP_KW_BIOLOGICAL_PROCESS (**B**) terms.

bioRxiv preprint doi: https://doi.org/10.1101/2023.05.02.539021; this version posted May 2, 2023. The copyright holder for this preprint (which was not certified by peer review) is the author/funder, who has granted bioRxiv a license to display the preprint in perpetuity. It is made available under aCC-BY 4.0 International license.

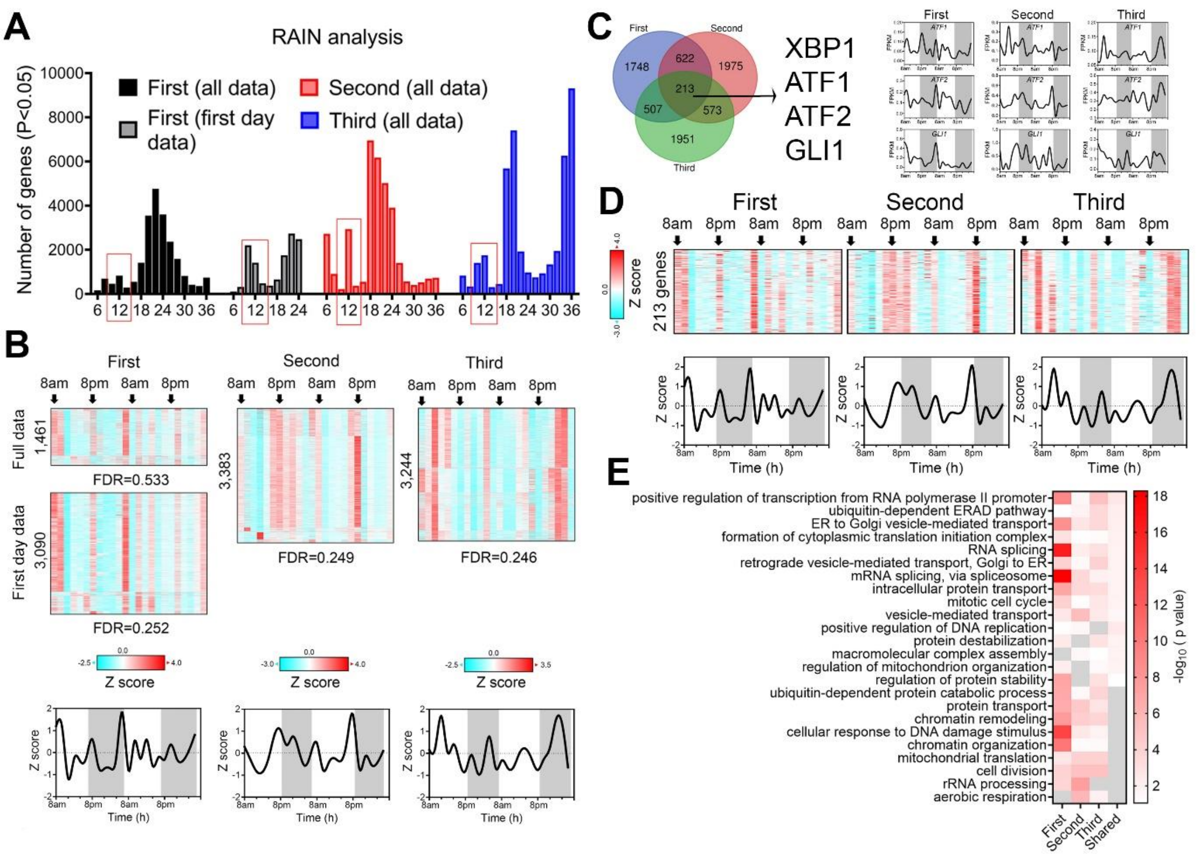

Fig. S6. RAIN analysis of the 48h temporal transcriptome. A single data point was inputted for each sample collected over the 48h protocol. **A**) Distribution of the number of genes cycling with different periods with p value smaller than 0.05. For the first subject, we further input the full 48 hours of data or the first 24 hours. (**B**) Heatmap and quantification of ~12h genes from all three subjects. The identification of a greater number of ~12h genes with lower FDR in the first subject with restriction of inputted data to the first 24 hours indicates the ~12h rhythm is dampened in the second day in this subject, consistent with what was found with the eigenvalue/pencil method as depicted in Figure S1G. (**C**) Venn diagram depicting common and distinct ~12h genes for each subject, with ATF1, ATF2 and GLI1 temporal expression shown on the right. (**D**) Heatmap and quantification of 213 common ~12h genes uncovered from all three subjects. (**E**) GO analysis of all and shared ~12h genes.

bioRxiv preprint doi: https://doi.org/10.1101/2023.05.02.539021; this version posted May 2, 2023. The copyright holder for this preprint (which was not certified by peer review) is the author/funder, who has granted bioRxiv a license to display the preprint in perpetuity. It is made available under aCC-BY 4.0 International license.

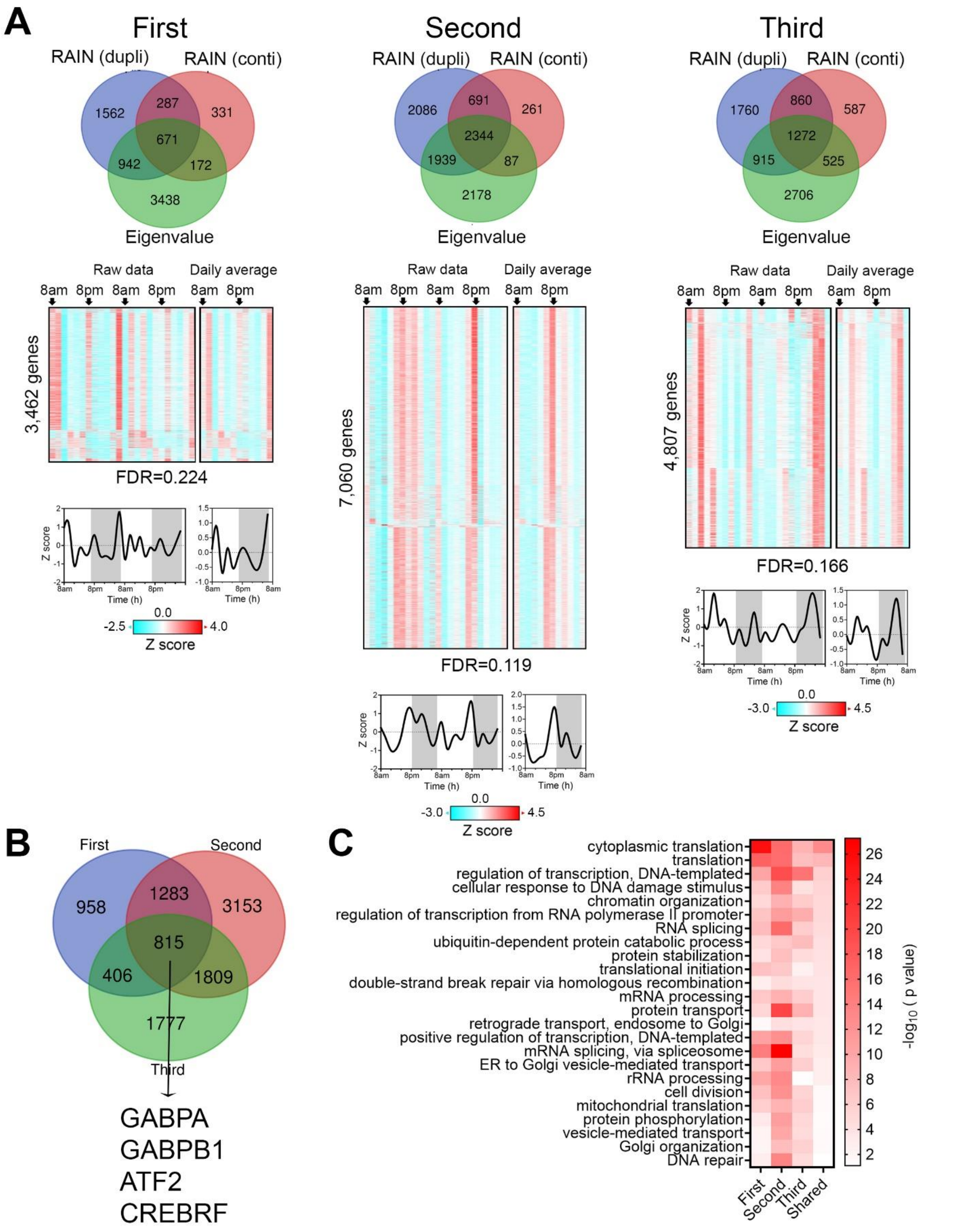

Fig. S7. RAIN analysis of the temporal transcriptome using consecutive 24h periods as biological replicates. (**A**) Venn diagram comparing the ~12h programs uncovered by the three

bioRxiv preprint doi: https://doi.org/10.1101/2023.05.02.539021; this version posted May 2, 2023. The copyright holder for this preprint (which was not certified by peer review) is the author/funder, who has granted bioRxiv a license to display the preprint in perpetuity. It is made available under aCC-BY 4.0 International license.

methods (top) and heatmap and quantification of ~12h genes uncovered by the RAIN dupli method in all three subjects (bottom). (**B**) Venn diagram depicting common and distinct ~12h genes uncovered in each subject. (**C**) GO analysis of all and shared ~12h genes in all three subjects.

bioRxiv preprint doi: https://doi.org/10.1101/2023.05.02.539021; this version posted May 2, 2023. The copyright holder for this preprint (which was not certified by peer review) is the author/funder, who has granted bioRxiv a license to display the preprint in perpetuity. It is made available under aCC-BY 4.0 International license.

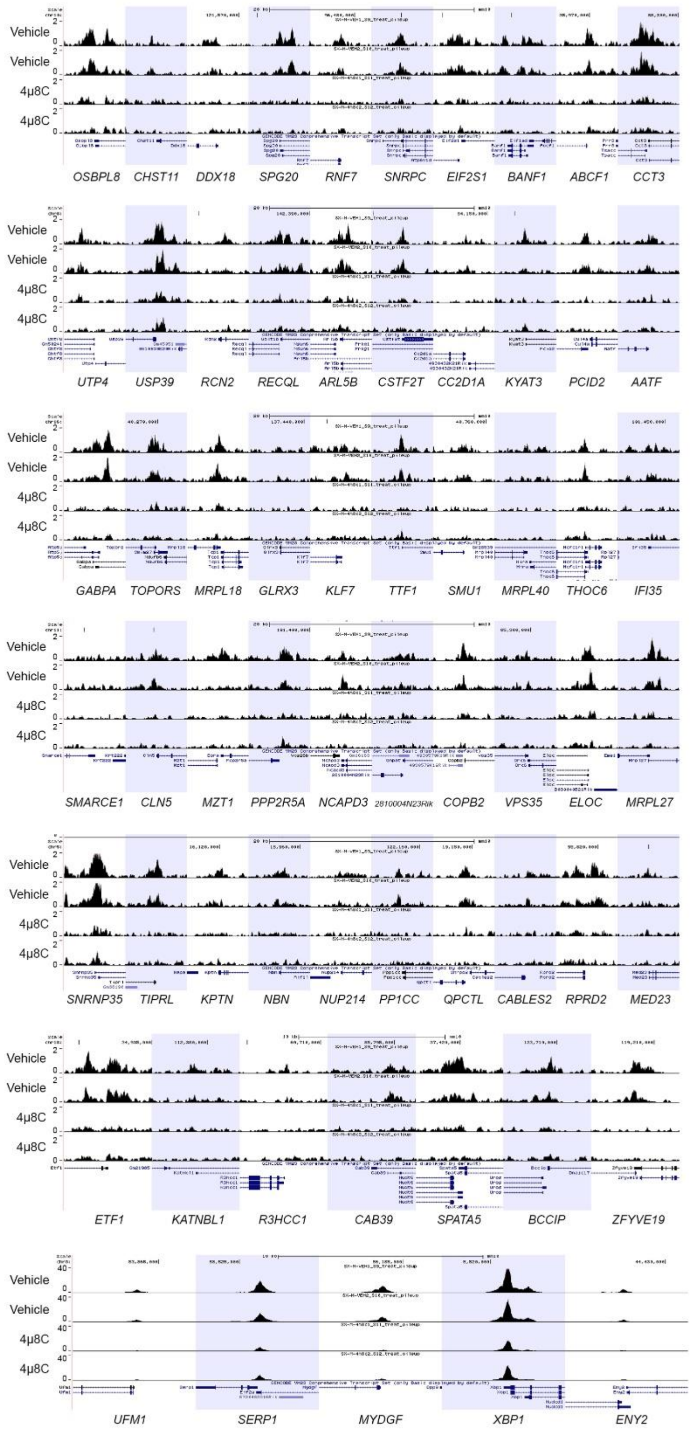

Fig. S8. XBP1 chromatin binding landscape of 12h genes in mouse Th2 cells. UCSC genome browser view of XBP1 chromatin recruitment to the promoters of orthologous 12h genes in murine Th2 cells.

bioRxiv preprint doi: https://doi.org/10.1101/2023.05.02.539021; this version posted May 2, 2023. The copyright holder for this preprint (which was not certified by peer review) is the author/funder, who has granted bioRxiv a license to display the preprint in perpetuity. It is made available under aCC-BY 4.0 International license.

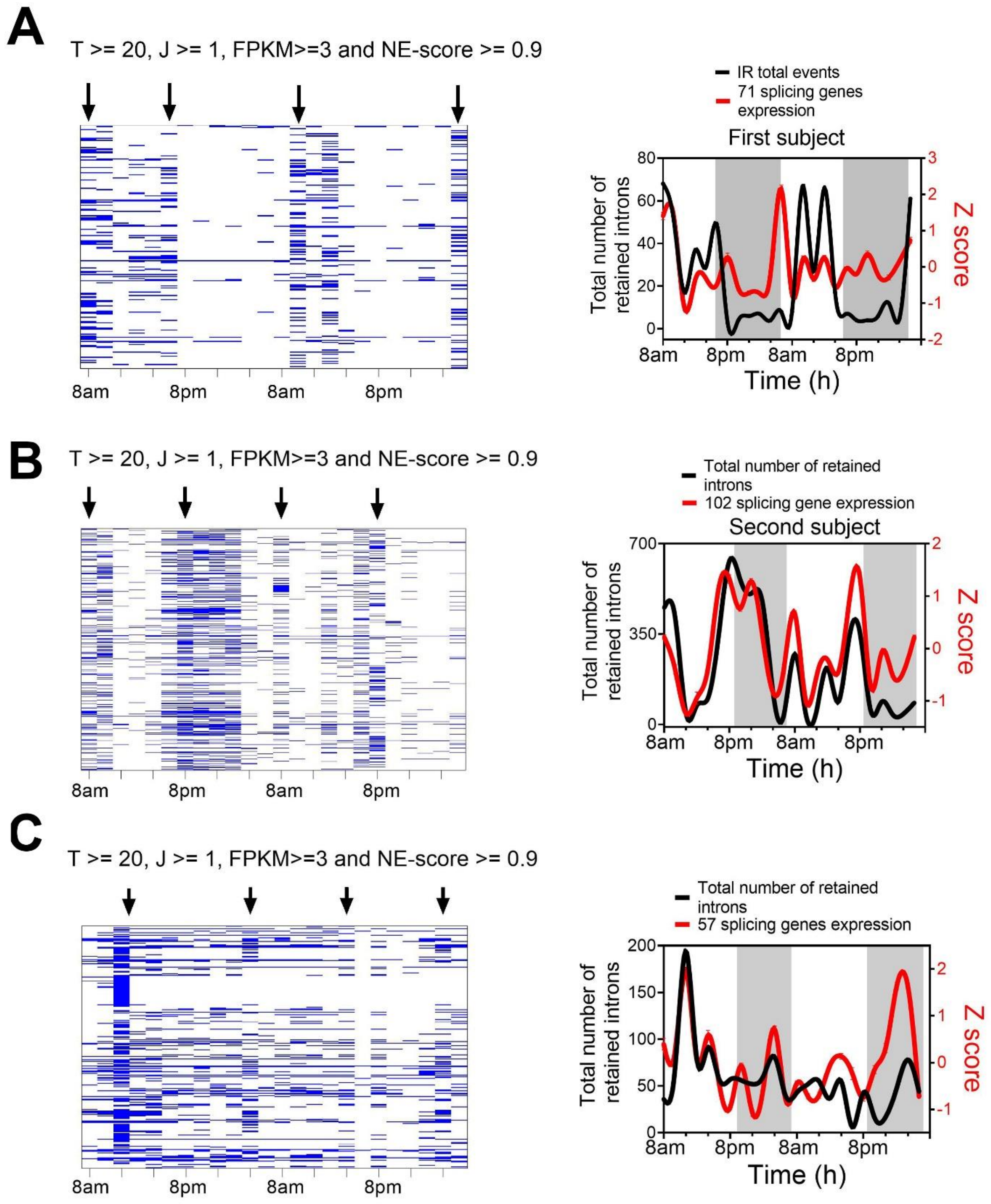

Fig. S9. ~12h rhythms are synchronized to global intron retention rhythms. (**A-C**) Heatmap (left) and quantification (right) of temporal IR events, superimposed with the Z score normalized temporal expression of ~12h splicing gene expression in the first (**A**), second (**B**) and third (**C**) subjects. IR events are detected with the more stringent setting of the iRead algorithm as described in the methods section.

bioRxiv preprint doi: https://doi.org/10.1101/2023.05.02.539021; this version posted May 2, 2023. The copyright holder for this preprint (which was not certified by peer review) is the author/funder, who has granted bioRxiv a license to display the preprint in perpetuity. It is made available under aCC-BY 4.0 International license.

A

GO of common retained introns in all three subjects

Term	PValue
T cell receptor signaling pathway	1.27E-06
antigen processing and presentation of peptide antigen via MHC class I	1.87E-05
positive regulation of T cell mediated cytotoxicity	3.64E-05
hemopoiesis	1.02E-04
protein autophosphorylation	2.99E-04
signal transduction	5.64E-04
immune response	7.32E-04
regulation of cell shape	8.10E-04
protein phosphorylation	8.49E-04
mRNA processing	9.56E-04
regulation of interleukin-1 beta production	0.00172
T cell differentiation in thymus	0.0026
regulation of tumor necrosis factor production	0.0026
actin filament capping	0.0031

B

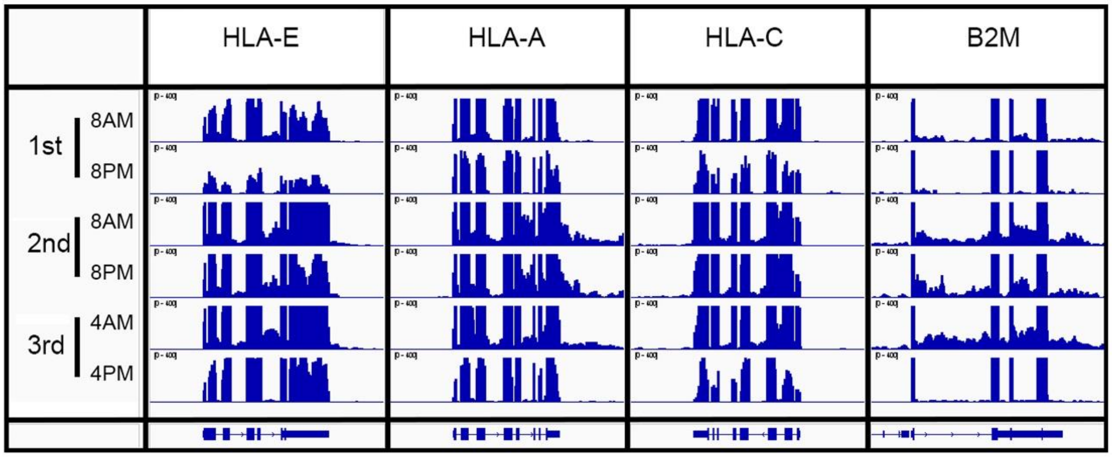

Fig. S10. Genes with intron retentions are enriched in antigen presentation genes. (A) GO analysis of common IR genes detected in all subjects. (**B**) Representative RNA-seq data demonstrating increased retained introns in MHC class I genes at 8AM or 4AM.

bioRxiv preprint doi: https://doi.org/10.1101/2023.05.02.539021; this version posted May 2, 2023. The copyright holder for this preprint (which was not certified by peer review) is the author/funder, who has granted bioRxiv a license to display the preprint in perpetuity. It is made available under aCC-BY 4.0 International license.

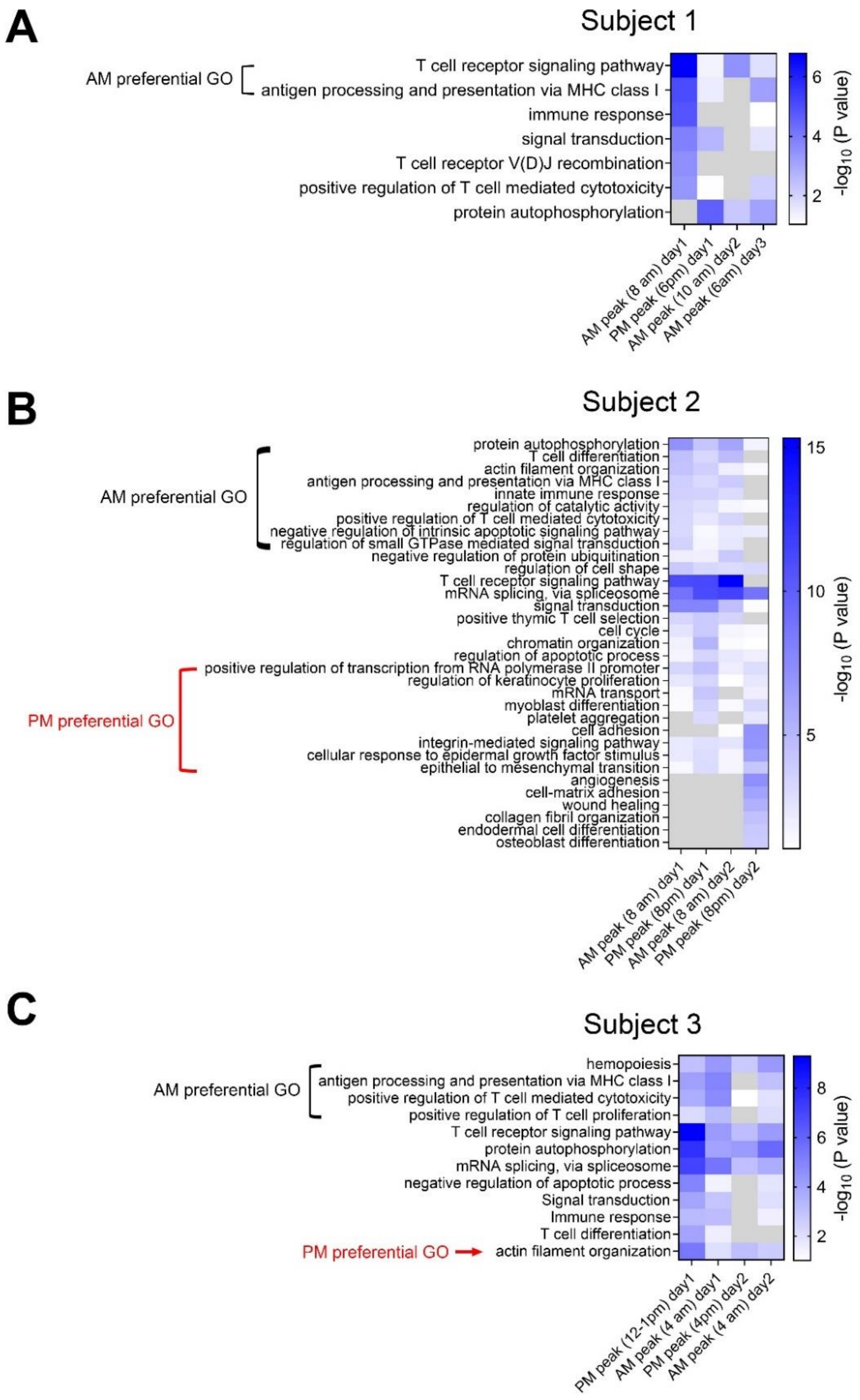

Fig. S11. Genes with intron retentions are enriched in immune genes. GO analysis of IR genes at different peaks in the first (**A**), second (**B**) and third (**C**) subject.
